# Determinants of Translation Elongation Speed and Ribosomal Profiling Biases in Mouse Embryonic Stem Cells

**DOI:** 10.1371/journal.pcbi.1002755

**Published:** 2012-11-01

**Authors:** Alexandra Dana, Tamir Tuller

**Affiliations:** The Department of Biomedical Engineering, Tel-Aviv University, Tel-Aviv, Israel; MRC Laboratory of Molecular Biology, United Kingdom

## Abstract

Ribosomal profiling is a promising approach with increasing popularity for studying translation. This approach enables monitoring the ribosomal density along genes at a resolution of single nucleotides.

In this study, we focused on ribosomal density profiles of mouse embryonic stem cells. Our analysis suggests, for the first time, that even in mammals such as *M. musculus* the elongation speed is significantly and directly affected by determinants of the coding sequence such as: 1) the adaptation of codons to the tRNA pool; 2) the local mRNA folding of the coding sequence; 3) the local charge of amino acids encoded in the codon sequence. In addition, our analyses suggest that in general, the translation velocity of ribosomes is slower at the beginning of the coding sequence and tends to increase downstream.

Finally, a comparison of these data to the expected biophysical behavior of translation suggests that it suffers from some unknown biases. Specifically, the ribosomal flux measured on the experimental data increases along the coding sequence; however, according to any biophysical model of ribosomal movement lacking internal initiation sites, the flux is expected to remain constant or decrease. Thus, developing experimental and/or statistical methods for understanding, detecting and dealing with such biases is of high importance.

## Author Summary

Gene translation is the process by which ribosomes translate mRNA molecules to proteins, a central process in all living organisms. Thus, understanding the biophysics of gene translation and the way its efficiency is encoded in the different features of the coding sequence has ramifications to every biomedical discipline. Recently, a new large-scale experimental approach named ‘ribosomal profiling’, has been developed for monitoring the ribosomal density at a resolution of single nucleotides. In this study, we analyzed ribosomal profiling data of mouse embryonic stem cells. These data enabled us to directly show that translation velocity is affected by the adaptation of codons to the tRNA pool, local mRNA folding of coding sequence, and local charge of the amino acids encoded in the coding sequence. In addition, our analyses suggest that ribosomal speed tends to be slower at the beginning of the coding sequence. Finally, we report possible biases in the ‘ribosomal profiling’ procedure that should be considered in future studies utilizing this method.

## Introduction

Gene translation is the second major step of gene expression and thus has ramifications related to every biomedical discipline including human health [1], [2], biotechnology [3], evolution [4]–[6], functional genomics [7], [8] and systems biology [9], [10]. One of the open questions in the field is related to the way translation efficiency is encoded in the transcript.

The most promising approach for studying gene translation is the ribosomal profiling method [11]. This approach was introduced only a few years ago but has already been successfully employed for answering various fundamental biological questions [12]–[19]. Specifically, ribosomal profiling has been used for: 1) understanding the mechanism of gene expression down-regulation by microRNAs [13], 2) understanding the dynamics of translation in mouse embryonic stem cells [12], 3) showing that the anti-Shine–Dalgarno sequence drives translational pausing and codon choice in bacteria [14], 4) studying the yeast meiotic program [15], 5) showing that miR-430 reduces translation before causing mRNA decay in zebrafish [17], and 6) to reveal the co-translational chaperone action of trigger factor in vivo [16].

In the current study we analyzed ribosomal profiles of mouse embryonic stem cells measured in a previous experiment [12]. The experiment output included ribosomal density measurements along hundreds of genes at a few time points, after preventing translation initiation. These data enabled us to infer the translation elongation speed in different genes, allowing us for the first time to study several biophysical aspects of translation elongation in mouse embryonic stem cells.

## Results

### Measuring translation elongation velocities

To study the kinetics of translation elongation in *M. musculus*, ribosome footprint profiles of isoforms expressed in embryonic stem cell were reconstructed based on a previous study [12]. Briefly, translation was halted by applying cyclohexamide. Fragments covered by ribosomes were mapped to the transcript and a baseline ribosomal read counts profile for each expressed isoform was created (see Methods). Let us denote these created profiles by 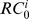, where 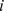 is the index of the analyzed isoform. In addition, to estimate the elongation speed of ribosomes, in three *additional* experiments harringtonine was used to stop translation initiation, while allowing ribosomes that already started translating the mRNA to continue their movement on it. Cyclohexamide was again applied 90/120/150 seconds after applying harringtonine to stop translation. In this work, the time difference between applying harringtonine and cyclohexamide for creating depleted profiles is named the ‘*run-off*’ time.

**Figure 1:**
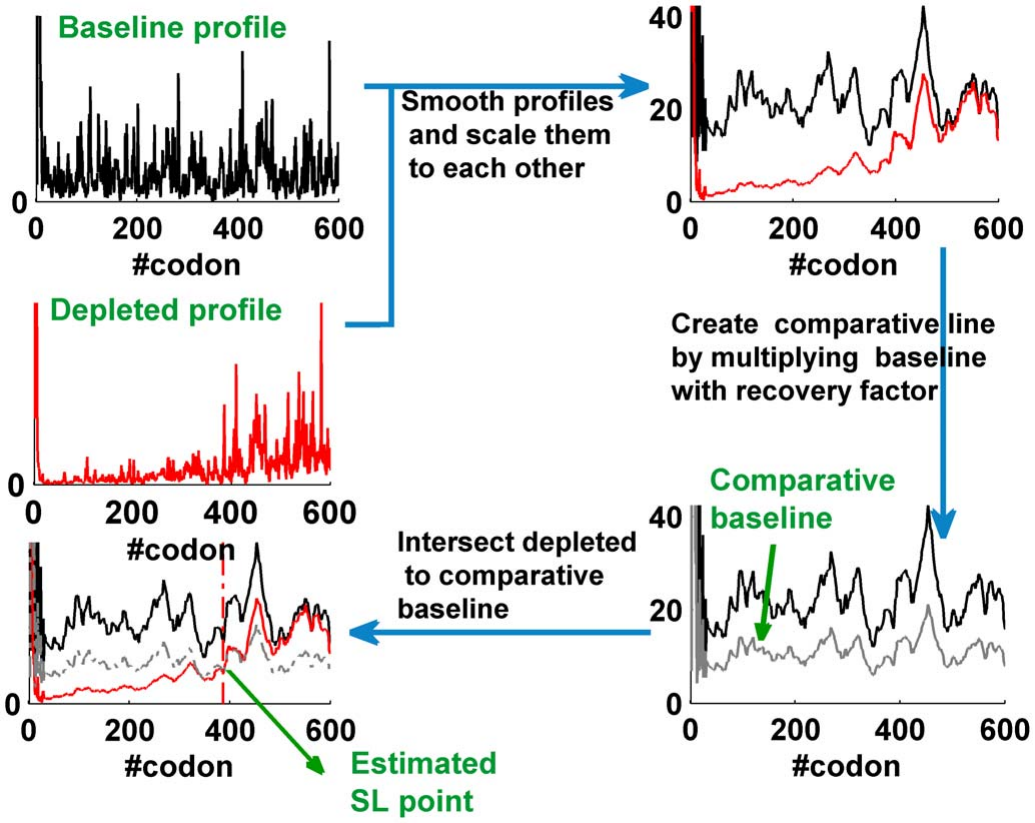
Estimating the *SL* point. A diagram outlining the methodology for estimating starting location points (*SL* points) demonstrated on the uc007gge.1 isoform. The black line depicts the ribosomal baseline profile while the red line represents a depleted profile (created using harringtonine to halt initiation; cychlohexamide was applied after 120 seconds). The dotted gray line represents the baseline profile multiplied by a recovery factor of 0.5, to which the depleted profile is compared. The red dotted line represents the estimated *SL* point.

Let us denote the ribosomal read counts obtained in each of these three experiments by 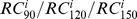 accordantly. The estimated **S**tarting **L**ocation of the depleted ribosomal profile (*SL*) was defined as the point where the ribosomal read counts profile of gene 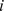 at time point 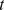 (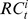 profile) reached half of the original ribosomal read counts profile 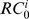 (Methods). Using these *SL* points, local translation elongation velocities were estimated for each analyzed isoform. Figure 1 outlines a schematic description of the method used to estimate the *SL* points, demonstrated on the uc007gge.1 isoform (see also Figure S7).

In the original work 4,994 isoforms with good read counts were found [12]. The authors noticed that the effect of harringtonine was best observed for genes longer than 750 codons, as for shorter genes the ribosomes managed to exit the mRNA for the used run-off times. Thus, only genes that were long enough (at least 750 codons) were used to infer the position of the *SL* points. In the current work, the same isoforms satisfying these conditions were analyzed, resulting in 785 processed isoforms (see Table S10). Let us define the three estimated *SL* points by 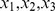 corresponding to time points 90/120/150 respectively. Let us mark with 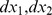 the segments defined by 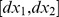, 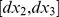 accordantly, and the ribosomal average translation velocity in these segments by 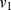 and 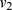. The average translation velocity of a segment was estimated by dividing the segments' length by 30 seconds. For each gene and time point, various quality checks were performed to reliably estimate the position of the *SL* points (see more technical details in the Methods section). Eventually, only isoforms with *SL* points satisfying 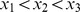 were selected, resulting in 692 valid isoforms out of the 785 processed isoforms (88%).

### Translation elongation speed varies among genes and tends to increase along the coding sequence

**Figure 2:**
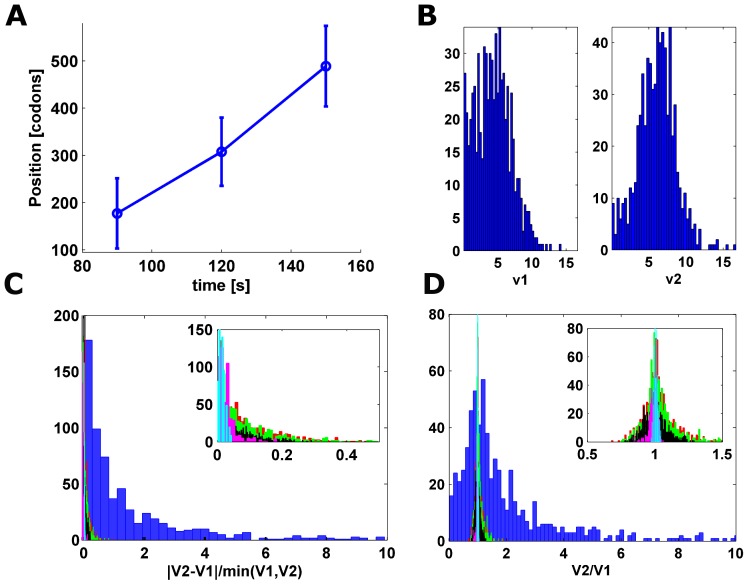
(A). Estimated position of the *SL* points (mean and standard deviation):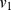 = 4.3+/−2.6, 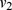 = 6+/−2.5. v = 5.2+/−1.2 (Wilcoxon test: p = 2.2*10^−26^). (B). 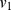 and 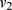 histograms. (C). Histogram of 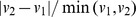 measure calculated on: 1) the experimental data (blue) (median value 0.82) 2) on simulated ribosomal densities of the analyzed isoforms for low/high/proportional initiation rates (green/red/black) and 3) ribosomal densities created using codons of equal translation efficiency for low/high initiation rates (magenta/teal). For the simulations, the obtained median values of the 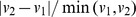 measures were 0.06/0.06/0.06/0.02/0.01, significantly lower than in the case of the experimental data (KS p-value <6.18*10^−153^ in all cases). The inset shows the ratio for the simulative data only. (D). Histogram of the 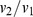 ratio calculated on real and simulative data. The median value of this measure for the real ribosomal profiles was 1.37, significantly higher than for the simulative data, which resulted in median values of 1/1.01/1.01/1/1.01 accordantly (KS p-value <5.67*10^−250^ in all cases).

Analysis of the data indicated that the median length of 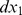 was 128 codons (130±77 codons) while the median length of 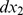 was 184 codons (181±75 codons). Therefore, although the mean translation velocity of all genes is around 5.5 *codons/second* [12] (see Figure 2A and Tables S4, S6), the average translation velocity along the second segment 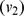 is larger than the average translation velocity along the first segment 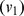 (6+/−2.5 *codons/second vs.* 4.3+/−2.6 *codons/second*, Wilcoxon test p = 2.2*10^−26^ Figure 2A). This result remains significant under various estimations methods of these velocities.

We performed additional analyses to support the conjecture that translation elongation velocity is not similar among genes: first, the standard deviation of the estimated *SL* points was between 17% and 49% (Figure 2A–B, Table S4, S6, columns 1, 2, 3). Second, the relative difference between the two estimated velocities (calculated using 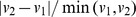) resulted in a median value of 0.82 while the median value of the ratio 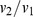 resulted in a value of 1.37 (see also Figure 2C–D). To compare the attained results to simulated genes with uniform translation elongation rate, we simulated 692 synthetic genes with 1) lengths distribution identical to the lengths distribution of the analyzed genes, and 2) with constant codons translation efficiency (see Methods). The ribosomal profile of these genes was simulated with a biophysical model (see Methods), resulting in a much smaller difference between the calculated velocities 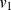, 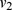 (median = 0.01; KS-test: p-value <1.81*10^−271^), as seen in Figure 2C. The ratio between the velocities 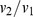 was also much more moderate when calculated on these simulated ribosomal profiles (0.99+/−0.03, KS-test p-value <1.56*10^−295^), as seen in Figure 2D. This comparison supports the claim that there is a high variance in the elongation speed of the analyzed genes.

### Estimated translation elongation velocity is significantly associated with features of the coding sequence

In order to explain the high variability among segments length, those were analyzed with respect to different features of the coding sequence, such as the adaptation to the tRNA pool (*e.g.* the tAI [20] and the CAI measure [21]), local mRNA folding energy [22] and local charge of the translated amino acids [22], [23]. Specifically, codons recognized by more abundant tRNA molecules increase the tAI measure, therefore we expect longer segments to positively correlate with this measure [24]. The CAI index, which measures the frequency of codons in a segment relatively to their appearance in highly expressed genes, is also expected to positively correlate with the segment length.

In addition, it was suggested that strong local mRNA folding tends to slow down ribosomal translation elongation as it increases the time it takes the helicases to unfold the mRNA molecules [24]. Therefore, segments more strongly folded (*i.e.* with lower folding energy (FE)) are expected to be shorter. Finally, the polypeptide must traverse two negatively charged regions to exit the ribosome [22], [23], [25], thus charged amino acids (specifically positively charged amino acids [23]) that are encoded in the codons preceding (upstream) the currently translated codon should have electrostatic interactions with the ribosome exit tunnel [22], [23], [25]. Therefore, segments more positively charged are expected to be shorter. More details about the calculation of these measures appear in the Methods section.

To estimate the distinct contribution of each of the coding sequence features to the elongation speed, we calculated the correlation between the length of the segments and each of these features, when controlling for the other two features, and after binning the data (details in the Methods section). Spearman correlation between the segments length and the genes' tAI/CAI when controlling for charge and folding energy of the segments resulted in a correlation coefficient of r = 0.29/0.21 (P<0.00615/0.049) accordantly. Spearman correlation between the segments' length and their mRNA folding energy when controlling for charge and gene tAI was r = 0.42 (P<4.72*10^−5^). The correlation between the segments' length and their charge when controlling for folding energy and the genes' tAI was r = −0.21 (P<0.046) (additional analyses appear in the supplementary). Thus, the results reported in the current subsection support the conjecture that the translation elongation speed is independently affected by each of the following features of the ORF: the adaptation of the ORF codons to the tRNA pools, local mRNA folding and local amino acids charge.

As mentioned in the previous section, the speed of translation elongation tends to increase along the coding sequence. Aiming at explaining this phenomenon, features measured on the first and second segment were also compared using a paired Wilcolxon test, resulting in significant values for folding energy (Wilcolxon test: P<1.04*10^−3^) but not for tAI/CAI and charge. This suggests that in mouse, a possible explanation of the increase in translation speed along the coding sequence is the decrease in the strength of the mRNA folding along the coding sequence. Finally, a weak but significant correlation between the average 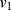 and 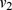 translation speed and the average transcripts length was observed in mouse (Spearman correlation: r = −0.05, p = 0.022), supporting the conjecture that shorter genes are more efficiently translated.

### Ribosomal flux inferred based on ribosomal profiling increases along the coding sequence, contradicting biophysical models of translation elongation

According to the accepted biophysical model of translation, during the elongation step ribosomes move along the coding sequence, translating each codon with a speed related to the features of the coding sequence in its vicinity and according to cellular factors such as concentrations of elongation factors and tRNA molecules. In addition, a ribosome may be delayed if a ribosome is located downstream in front of it [26]. It is also assumed that in general, ribosomal abortion during translation is relatively rare and that initiation usually occurs at the 5′UTR (*i.e.* ribosomes do not appear in the middle of the coding sequence [26]).

**Figure 3:**
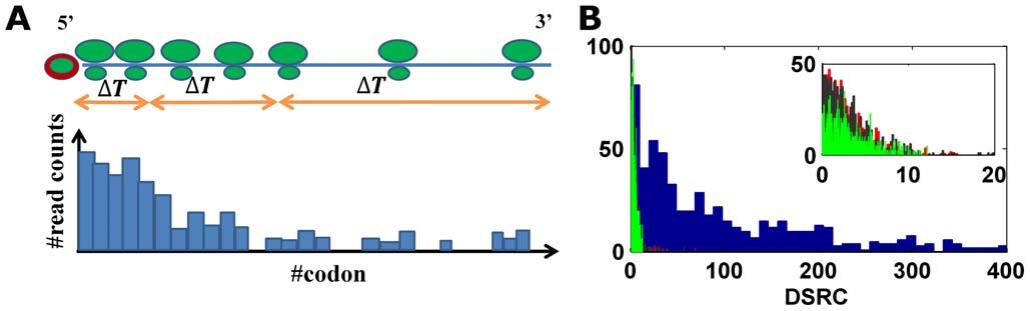
(A). Ribosome read counts measures according to the biophysical model. The green round shapes represent the ribosomes on the mRNA, which is depicted with a blue line. According to the biophysical model, segments of high ribosomal read counts are associated with regions more slowly translated (bottom graph). The orange double arrows represent the mRNA segments being translated in equal time intervals. (B). 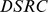 histogram calculated on real (blue) and simulated ribosomal profiles for low/high/proportional initiation rates (black/red/green) with zero noise level. The calculated median value of this measure is 88/2.46/2.39/2.38 accordantly.

According to the protocol of the experiment (*e.g.* see [11], [12]), ribosomal footprint reads of a certain codon are generated when the codon is covered by ribosomes. From a biophysical perspective, slower codons are covered by ribosomes for a larger amount of time (relatively to other codons in the mRNA), creating a higher number of reads (for an illustration see Figure 3A).

In this study, for each analyzed isoform, both 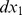 and 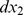 segments were assumed to be translated in an equal time interval of 30 seconds, therefore according to the above assumption, on average, it is expected for the sum of read counts in the 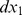 and 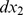 segments (measured on the baseline profile 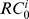) would be equal. Therefore, in each isoform the shorter segment is expected to have a higher ribosomal read count per nucleotide in comparison to the longer one.

Let us mark the **s**um of **r**ead **c**ounts in intervals 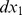 and 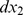 by 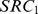, 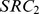 accordantly. Let us define the percentage **d**ifference between 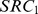 and 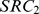 (relatively to the minimum of 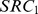 and 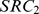) by
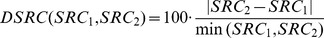
This measure is invariant to the genes' various mRNA levels and translation initiation rates, therefore enabling comparison between all analyzed isoforms. Using the above assumption, we expect this measure to be close to zero. Figure 3B shows the histogram of the 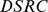 measure calculated both on the real ribosomal profiles and on the simulated ribosomal profiles created using the TASEP biophysical model for various initiation rate values (see Methods). However, in contrast to the made biophysical assumptions, the results indicate that for a substantial part of genes, the 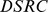 measure is abnormally high (median value of 88 *vs.* 1–6 for simulative data of different levels of noise; KS test, all p-values <3.97*10^−215^).

In addition, the ribosomal flux at a certain codon 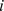 along the coding sequence is defined as the multiplication of the translation velocity and density at this point 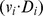. Therefore, according to any biophysical model with negligible amount of initiation events inside the ORF, we expect the flux to be constant (*i.e.* 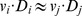 for different 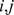) or decrease (due to ribosomal abortion);

Let us mark the mean ribosomal read counts measured in the first and second segments by 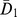 and 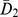 respectively and the average velocity in the first and second segment by 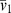 and 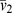. If we assume that the local flux remains constant, we also expect that 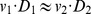. Given that the average velocities of 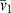, 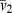 in both the first and second intervals were measured during the *same* time intervals, we can rewrite this relation as 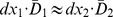


**Figure 4:**
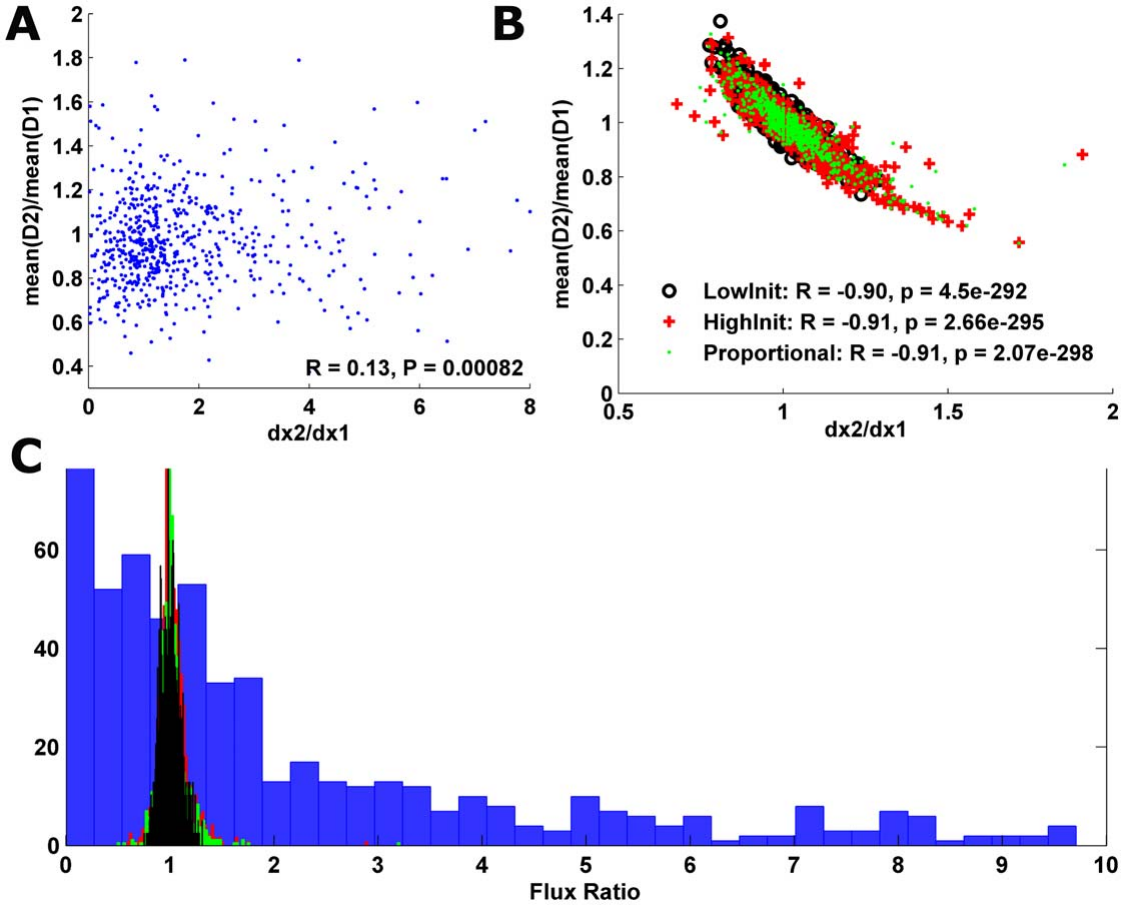
(A).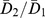 ***vs.*** 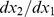 **measured on the real data (B).** 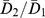 ***vs.*** 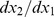 **measured on the simulative data for low/high/proportional initiation rates (black circles/red crosses/green dots) (C). Histogram of the flux ratio** 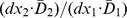 measured on the real (blue; median = 1.69) and on the simulative data created using low/high/proportional initiation rates (black/red/green; median = 1/1.01/1.01; KS test: p-value <9*10^−95^).

Thus if
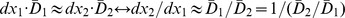
we would expect the correlation between 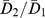 and 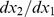 to be *negative*. Intuitively, for a given gene, longer segments should have relatively lower mean read counts. Indeed, the calculated ratios for the simulated densities resulted in a negative correlation (Figure 4B, Spearman correlation of R = −0.9, P<10^−291^; R = −0.91, P<10^−294^; R = −0.91, P<10^−297^; for low/high/proportionate initiation rates). However, when measured on real ribosomal read counts profiles, the correlation between 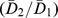 and 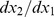 achieved a significant *positive* value (R = 0.13, P<0.00082; Figure 4A), contradicting the accepted translation model. Finally, the flux itself 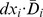 is expected to remain constant or decrease (due to ribosomal abortion), *i.e.* 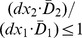. Yet, we found that this ratio tends to *increase* (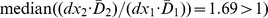.

Next, we calculated the values of all presented measures on the simulated ribosomal profiles for different initiation rate regimes (see Methods) and compared them to the values obtained when calculating them on the real ribosomal profiles. This analysis resulted in significantly different values: the 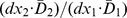 measure calculated on the simulative data resulted in a median value of 1.01 (KS test in comparison to the measured data: p-value <9*10^−95^; Figure 4C, Table S9), while the difference between the velocities 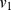 and 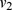 resulted in a median value of 0.06 (KS test: p-value <6.18*10^−153^; Figure 2B). In addition, the ratio between the velocities resulted in median values of 1–1.01 (KS test: p-value <5.67*10^−250^) (Figure 2C). Overall, the comparisons between all measures calculated on the experimental data and on the simulative ribosomal profiles created by the biophysical model point on the existence of substantial biases in the data produced by the ribosomal profiling procedure.

## Discussion

In this study, we reanalyzed the ribosomal profiling data of mouse embryonic stem cells that was generated in a previous study [12]. Our analysis demonstrates that even for relatively long analyzed genes, that are not expected to be under strong selection for translation efficiency [27], in unusual tissue/conditions such as embryonic stem cells, translation elongation speed is affected by features such as the adaptation of codons to the tRNA pool, local mRNA folding, and charge.

In addition, our analysis directly shows for the first time that the translation elongation speed tends to increase along the coding sequence. The reasons for this phenomenon may be related to the fact that at the beginning of the coding sequence features such as adaptation to the tRNA pool and mRNA folding strength tend to slow down ribosomal movement (see, for example, [22], [24]). This may also be related to the fact that there is a selection for lower codon bias at the beginning to reduce the costs of both missense and nonsense translational errors [28], [29]. The statistical analysis performed in this study support the conjecture that the slower speed at the beginning of the coding sequence is due to stronger mRNA folding in this region. This phenomenon, however, may also be related to yet unknown properties of this process or to biases of the ribosomal profiling methods.

Finally and importantly, at least in the reported study, our analysis demonstrates the existence of some unexplained deviations between the output of the ribosomal profiling approach and any of the accepted models of translation elongation, which assume that the rate of initiation from sites inside the ORF is negligible. This discrepancy may be explained by the fact that current models of translation elongation are inaccurate and, for example, initiation does tend to occur from sites inside coding sequences. However, the most plausible explanation is that ribosomal profiling approach, as in the case of the more traditional approaches for studying mRNA levels (*e.g.* [30]), includes experimental biases that should be further explored. Another bias of the ribosomal profiling approach which is related to the increased ribosomal density at the beginning of the ORF has been suggested recently in [12].

We also suggest a few explanations for these observed biases, while taking into consideration that there might be additional sources of bias in the ribosomal profiling protocol that are not mentioned here. For example, an insufficient number of mRNA molecules could increase the estimation errors and bias all the presented measures. Specifically, the ribosomal profiling approach produces for each gene the ribosomal positions along the mRNA molecules that have been transcripted from it and that are present in the cell at the time of the experiment. As the read counts per location of a single mRNA are stochastic, averaging them over many mRNA molecules of a gene should theoretically produce a profile that is similar to the stationary density profile of the gene. Thus, the number of mRNA copies affects the averaged profile and eventually the quality of the estimated measures mentioned in this study. In practice, genes with a relatively low number of mRNA molecules can result in highly biased profiles. Indeed, when we modified our computational simulation of the experiment to simulate a low number of mRNA molecules per gene (see Methods), the correlation between 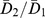 and 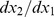 decreased (Figures S12, S13, S14) while the *DSRC* measure increased (Figures S9, S10, S11), contrary to the expected trend.

Another source of bias may be related to the fact that the current ribosomal density protocol involves filtering some of the reads, distorting the resultant ribosomal density profiles. Specifically, by the protocol of the experiment, only short mRNA fragments that are covered by exactly one ribosome (*i.e*. monosomes) are purified for further analyses [11], [31], while mRNA segments covered by polysomes are discarded. Thus, it is also possible that the reported biases are, at least partially, due to the fact that fragments that origin from ribosomes located very close to each other on the mRNA are filtered and not analyzed, creating deviated ribosomal profiles. Indeed, cases of fragmented mRNA covered with more than one ribosome as a result of very close ribosomes were reported in a previous study [32]. In addition, when only monosomal footprints were considered in the simulation (see Methods), we obtained a decrease in the correlation between the 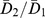 and 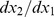 ratios and a major increase in the *DSRC* measure (see Figures S9, S10, S11, S12, S13, S14).

The deviations from the accepted biophysical model could also be explained by the non-uniform effect of the harringtonine/cyclohexamide substances on the different mRNA molecules, causing uneven run-off times, and distorting the location of the *SL* points. The simulation of this possible experimental bias (see details in Methods) also resulted in an increased *DSCR* and a decrease in the correlation between 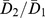 and 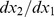 (Figures S15, S16, S17).

Finally, complex relations between the sequence features, their effect on ribosomal density and on the output of the ribosomal profiling approach may also contribute to the deviation from the biophysical model. For example, it was suggested that elongation speed and ribosomal density are affected by the strength of the local folding of the mRNA (stronger folding→slower elongation speed→high ribosomal density) [22]. However, it is also possible that stronger mRNA folding decreases the efficiency of footprint production in the ribosomal profiling protocol (*e.g.* the efficiency of RNase activity decreases for mRNA fragments with strong folding; *e.g.* see [33]), contributing to a distorted ribosomal density profiles.

Nonetheless, currently, the ribosomal profiling approach is the major method for studying gene translation, therefore understanding these biases and accurately correcting them should significantly affect studies in various biomedical disciplines. As was demonstrated in this study, one possible direction for detecting such biases is by comparing the ribosomal profiling outcome to the computational biophysical models using statistical analysis. We believe that such approach will be used in the future for employing filters and normalization procedures that are inversed to the noise/bias obtained in the experimental procedure and for adjusting the experimental procedure itself.

## Methods

### Reconstructing the genes' ribosomal profiles

Sequencing data were downloaded from the GEO database (accession number GSE30839) [12]. We analyzed all data related to the study of the kinetics of translation elongation. The specific processed files are summarized in Table S1.

Sequenced reads comprise short RNA fragments of different lengths; therefore, a generated linker sequence (CTGTAGGCACCATCAATTCGTATGCCGTCTTCTGCTTGAA) was attached to enable the recovery of the original fragment. More details of this method appear in the original work [12]. In this study, linkers were first detected and removed from the published fragments and only then aligned to transcripts. The start location of the linker was estimated to be between the 20–36 *nt* of the RNA fragment. Next, the distance between the estimated linker and the published linker was calculated (in terms of number of different nucleotides); a valid linker was accepted if this distance differed by up to two nucleotides. If no valid linker was found, the fragment was rejected. Table S2 summarizes the number of fragments published by Ingolia *et al.* (see Table S1, column 2) and the percentage of processed fragments after removing the attached linker (column 3).

Aligning the fragments directly to the genome resulted in a high number of ambiguous matches. Therefore, fragments were aligned to known transcripts (exons) and spliced junctions. The *M. musculus* transcripts were derived from the UCSC Genes data set [34] and the alignment was performed using the Bowtie software [35], allowing up to two mismatches.

As mentioned by Ingolia *et al.*, fragments of different lengths tend to have slighter different A site locations, therefore the beginning of the A site for fragments of 29–30/31–33/34–35 nt was defined to begin +15/+16/+17 *nt* relatively to the 5′ end of the fragment. Additional details about this topic appear in the original work [12].

As summarized in Table S2, part of the processed fragments matched to more than one location. To overcome multiple mapping of a single fragment, we performed the following procedure: first, only fragments aligning to a single location were mapped. In the second iteration, for all fragments aligning to more than one location, the mean read counts in the region of the possible locations was calculated (10 *nt* before and after the location of the A site for each possible location). These mean read counts defined the probability of an ambiguous fragment to be aligned to only one of the locations.

For each isoform, nucleotide read counts profiles were reconstructed by assembling read counts of relevant exons and spliced junctions. Codon reads were calculated by averaging the obtained reads of each three non-overlapping consecutive nucleotides.

### Estimating the position of the ribosomes at each time point by the original method

In the *original* work, the 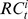 profiles were smoothed using an averaging window of five codons and normalized by the average read counts of codons 800–1000. This normalization assumed that read counts in regions not affected by harringtonine (codons 800–1000) have a similar value (for each one of the run-off profiles apart). When assuming the experiment is reproducible, *i.e.* ribosomal read counts of all 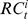 profiles are similar after the first 750 codons (the harringtonine effect did not extend beyond this point for any isoform in the experiment [12]), it is possible to estimate the **S**tarting **L**ocation (*SL*) point of a depleted profile 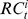 by comparing it to the baseline profile, 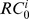. The *SL* of the depleted ribosomal profile of each isoform was defined as the position beyond the first 40 codons, where the normalized ribosomal density profile 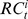 exceeded a value of 0.5. In this work, this parameter is defined as the recovery factor. Isoforms with *SL* points not satisfying 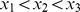 (see Table S3) were discarded. When smoothing the profiles with longer averaging windows, the number of isoforms with non-physical *SL* points reduced to 141 (out of 785, see also Table S3).

### Estimating the position of the ribosomes at each time point by the new method

Further study of the nature of ribosomal profiles revealed that the original *SL* estimation method suffers from some difficulties: the results presented in Figure S3-A show that read counts in regions not affected by harringtonine (beyond the 750^th^ codon, excluding the last 20 codons) have a high variability, therefore their average read count value cannot be used for normalizing the ribosomal profiles. In addition, in the original method the *SL* point was defined as the location where the run-off profile exceeded the threshold value 0.5. This criterion assumes again that 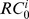 profiles are relatively homogenous, and small spikes caused by noises can be filtered by first smoothing them. However, the results in Figure S3-B show that different profiles have a high read counts variability, also suggesting that ribosomal read counts could be position dependent, making the comparison of the run-off profile to a static threshold of 0.5 problematic.

To overcome these issues, in the current work we suggested scaling each run-off profile to the baseline profile by a dynamic factor that derives from the read counts beyond the 750^th^ codon of both profiles (excluding the last 20 codons). This factor is set to minimize the distance between these regions. In the current study, we also tested the effect of the smoothing window size (10/15/20/25/30 codons) on the number of genes with physical *SL* points, as presented in Table S5. The *SL* location of each isoform was defined as the position beyond the first 40 codons, where the ribosomal density profile 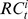 exceeded the value of the 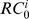 profile multiplied by the recovery factor. This created a dynamic threshold for the run-off profiles to be compared to. The influence of the recovery factor on the number of genes with physical *SL* points was also evaluated, as presented in Table S6. In addition, to improve robustness of the method to local bursts of noise, an *SL* point was defined to be valid if 50% of the next 20 points could also exceed the dynamic threshold. The optimal smoothing window size and recovery factor were selected to maximize the number of genes whose *SL* points were physically estimated (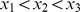), resulting in a window size of 30 codons and a recovery factor of 0.5 (see Table S3, S4, S5, S6).

To compare between the methods' ability to correctly estimate *SL* points in a noisy environment, both the original [12] and the newly suggested methods were also evaluated on synthetic data created using the TASEP model (*e.g.* see [22]). *SL* points were estimated for different run-off times and different levels of additive noise (see Methods, evaluating the error rate of the *SL* points). Figures S4, S5, S6 show the mean and standard deviation estimation error as function of noise level and size of the smoothing window for both estimation methods. As seen from the results, on the simulative data the newly suggested method achieved a lower estimation error for all levels of noise and smoothing window sizes.

For comparison, in this work, the various tested measures were calculated based on *SL* points estimated using both methods. The smoothing window size was set to 30 codons and the recovery factor was set to 0.5. The figures in the main text were generated using the new method with these parameters. More details appear in Text S1.

### Calculating the average folding energy of a segment

Folding energy (FE) of a nucleotide was defined as the folding energy of a 40 *nt* segment, starting from the current nucleotide. The segment's FE was calculated using the rnafold Matlab function [36]. The FE of a gene (segment) was defined as the average folding energy of its nucleotides.

### Calculating the average tAI measure of a segment

Codon tAI values were calculated according to [20], using tRNA copy numbers published in http://gtrnadb.ucsc.edu/Mmusc10/. The tAI value of a segment was calculated using:
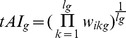
Where 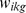 is the relative adaptiveness of codon of type 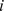, 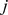 the index of the codon and 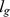 the number of codons in segment 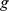. Let 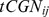 be the copy number of the 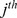 anti-codon that recognizes the 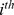 codon, and let 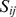 be the selective constraint of the codon/anti-codon coupling efficiency. Then, the absolute adaptiveness value of a codon is defined by
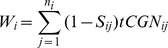
The relative adaptiveness value of a codon 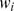 is obtained by normalizing 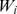 with the maximal 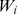 value among its 61 values (for specific values see Table S10).

### Calculating the average CAI of a segment

To calculate CAI of a segment, codons were ranked according to their usage in ribosomal proteins 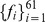 (). Using these frequencies, the CAI of a segment was similarly defined in the following manner:
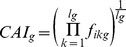



### Calculating the average charge measure of a segment

For each gene, a vector of charges was defined by assigning +1 to positively charged amino acids (Arg and Lys) and −1 to negatively charged amino acids (Asp and Glu). The charge of other amino acids was set to 0. A sliding window of 40 codons was applied on the charge vector to smoothen the charge effect on the mRNA. The overall charge of a segment was defined as the sum of its charges.

### Simulating ribosomal densities

To enable analysis of various features in a simulated environment, ribosomal densities of the analyzed isoforms in this work were calculated using the TASEP biophysical translation model, previously used in different studies (*e.g.* [22], [37]). The mRNA was modeled using a lattice of 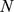 sites, representing the number of codons of the isoform. Each ribosome was defined to cover 11 codons and the A site was located at the sixth codon. During translation, any codon could be covered at a time by a single ribosome at most. In each step of the simulation, a single ribosome was allowed to attach itself to the lattice or advance to the next codon if the first/next six codons were not occupied. The time between initiation attempts was set to be exponentially distributed with a constant rate 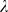. Similarly, the time between jump attempts from site 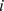 to site 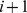 was assumed to be exponentially distributed with rate 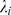.

The time between events, (initiation or jumping between sites) is therefore exponentially distributed (minimum of exponentially distributed random variables) with rate:
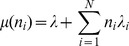
where 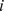 describes the site (codon) number on the lattice and 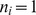 if codon 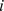 is being translated, otherwise 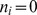. Therefore the initiation probability is given by 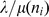 and the probability of a ribosome to jump from site 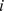 to 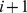 is given by 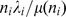.

The 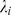 parameter was determined for each codon type according to its translation efficiency, estimated by the tAI measure (for specific values see Table S10). The initiation rate 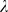 was studied for different values, depicting different initiation rate regimes.

To achieve an initial scattering of the ribosomes on the mRNA, 10^6^ simulations steps (events) were performed. This number of steps was selected to enable full initial steady state ribosomal cover for the analyzed genes. In general, longer genes or genes with low initiation rates (relative to the gene's codon translation efficiencies) require a higher number of simulation steps to achieve this condition.

To calculate ribosomal density profiles, we simulated another 10^7^ steps. In each step, the simulation updated the time each site was translated by a ribosome. The final vector of times representing the total time a site was translated by a ribosome was then normalized by the total time of the simulation. In addition, the final scattering location of the ribosomes on the mRNA was saved.

### Simulating ribosomal densities for different run-off times

Simulated ribosomal profiles were created by using three different initiation rate regimes 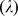: low, high and proportional to the genes' mean ribosomal read counts. The low initiation rate was set to be 10% of lowest codon translation rate (based on the tAI measure), while the high initiation rate was set to be twice the value of the highest codon translation rate (based on the tAI measure).

Proportional initiation rates were set for each isoform according to its measured mean ribosomal read counts (excluding the first 60 and last 40 codons). This initiation rate type assumed that in general, genes with higher mRNA and ribosomal densities levels (thus higher ribosomal read counts) are more highly expressed, therefore their initiation rate should be higher. Thus, for this regime initiation rate of the isoform with the lowest mean read counts was set as half of the slowest codon translation rate, while the initiation rate of the isoform with the highest mean ribosomal read counts value was set to twice the value of the highest codon translation rate. Initiation rates for the rest of the genes were set with equal distance between these two extremes, according to the genes' mean ribosomal read counts.

To simulate ribosomal profiles for different run-off times, the TASEP model was run 10^6^ simulations steps to achieve a steady state ribosomal spread on the mRNA. Initiation halting was simulated for 100 different run-off times, defined by
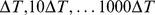
where 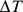 was defined to be the maximal translation time of a codon (based on the tAI measure).

To simulate numerous mRNA copies per gene, for each run-off time and analyzed gene, 500 ribosomal density profiles were calculated and those were averaged with equal weight to obtain a representative ribosomal profile for each gene and run-off time. More details appear in Text S1.

### Simulating ribosomal densities for different run-off times for genes with codons of equal translation efficiency

In the original work, it was claimed that translation elongation is constant throughout the translation of the mRNA. To test this hypothesis, we created synthetic genes using the length of the analyzed genes in this work, but with codons of equal translation efficiency, which was set as the mean tAI value of the codons calculated in *M. musculus*. Using the TASEP model, the ribosomal profile of each one of the synthetic genes was created for different run-off times 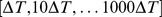 for low and high initiation rates. More details appear in Text S1.

### Evaluating the error rate of methods that estimate *SL* points

To allow accuracy evaluation of the original and new method for estimating *SL* points, ribosomal density profiles with specific run-off times were created, as previously described. To test the robustness of the estimation method for different levels of noise, additive uniformly distributed noise of different levels was added prior to estimating the *SL* points of each analyzed gene. The noise level added to each gene was selected to be proportional to its maximal *simulated* ribosomal density, such that
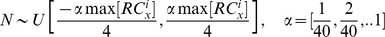
Let us mark by 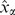 the estimated *SL* location for a noise level characterized by 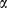. The estimation error is then defined by
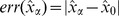
The *SL* points for all simulated genes for run-off times of 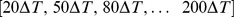 were calculated for the above noise levels. The general estimation error for a given noise level was defined as the average estimation error for all tested genes and run-off times. More details in Text S1.

### Calculating different measures on the simulated ribosomal densities

For each simulated ribosomal profile (based on the real analyzed genes) and various initiation rates (low/high/proportional) the estimated *SL* points were calculated for run-off times of 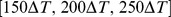. These points were selected to resemble the real aggregated profiles (see Figures S1, S2).

These *SL* points were used for calculating the ratio between the estimated velocities 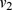 and 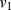, analysis of the 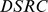 measure and correlation between the ratio of the mean read counts and the ratio of the segments length.

In addition, these measures were also calculated for the simulated ribosomal profiles of genes composed of codons with equal translation efficiency (same run-off times as described above), for low and high initiation rate. More details appear in Text S1.

### Simulating the influence of removing fragments covered by polysomes on the obtained ribosomal densities

To simulate ribosomal densities profiles obtained after filtering long fragments (created by adjacent ribosomes), for each simulated mRNA copy, ribosomal read counts were considered only for fragments covered by ribosomes that had a least one codon gap between themselves and their neighboring ribosomes, on both sides (using the final ribosome scattering on the mRNA). More details appear in Text S1.

### Simulating non-uniform effect of harringtonine

To simulate a non-uniform effect of the propagation time of harringtonine, the analyzed isoforms were simulated using the TASEP model for low initiation rate (this regime results in profiles similar to the real measured profiles, see Figure S1, S2). For each gene, initiation halting was calculated for the following run-off times 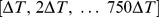 when using 500 mRNA copies per gene. Let us denote the ribosomal profile of gene 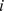 calculated for the mRNA copy 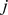 and run off time 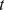 by 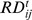 Let us denote the aggregated profile of gene 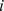 for the run-off time by 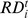. The non-uniform effect of harringtonine was simulated for each gene by aggregating different run-off profiles is the following manner:
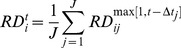
Where 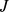 is the number of mRNA copies simulated per gene and 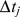 is a random variable, such that 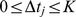. The simulation was calculated for 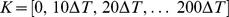. The higher the 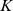 value, the more prominent the effect of the non-uniform propagation time of haringtonine. More details appear in Text S1.

### Calculating correlations between various measurements and computation of p-values

The comparison between the translation velocity 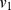 and 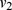 was done using the paired Wilcoxon test, as supplied in the Matlab 2011b software. The comparison between the 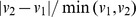, 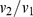, 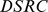, 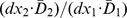 measures, calculated on the real ribosomal profiles and on the simulated ribosomal profiles, was done using the two samples Kolmogorov-Smirnov (KS)-test. The correlation between the segments' length and their tAI/CAI/FE/charge properties was calculated using partial Spearman correlation, as supplied in the Matlab 2011b software. The comparison between the translation velocities of segments with top/bottom 20%–50% of the tAI/CAI/FE/charge properties that appear in the supplementary results was calculated using the unpaired t-test and the two samples KS-test. The value of the tAI/CAI/FE/charge in the first and second segment (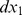 and 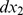) was also compared using a Wilcolxon test. The correlation between the tAI/CAI and gene length was calculated using Spearman correlation.

Before we performed partial correlation between tAI/CAI/FE/charge measurements and segment length we binned the data in the following manner: first, segments were sorted according to their length and then divided into bins of 15 samples. For each bin, the average length/tAI/CAI/folding energy/charge was calculated in order to reduce noise.

## Supporting Information

Figure S1 **Reconstructed ribosomal profiles using real fragments, for different run-off times – average view.**
(TIF)

Figure S2 **Simulated ribosomal profiles for different run-off times – average view.**
(TIF)

Figure S3
**Histogram of the normalized standard deviation (STD) calculated for genes with good reads and with at least 1000 codons.** The standard deviation was calculated using the real ribosomal profiles 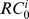 (blue) and based on simulative profiles created using the TASEP model. We considered different initiation rate regimes for the TASEP - low (red), high (black) and proportional (green). (A.) Normalized STD calculated on read counts of codons 730–1000. (B.) Normalized STD calculated on read counts of all codons, except for the first 40 and last 20 codons.(TIF)

Figure S4 **Estimation errors of the old (red) and the newly suggested estimation method (blue) as function of different noise levels, created with a TASEP simulation with** ***low*** **initiation rates.**
(TIF)

Figure S5 **Estimation errors of the old (red) and the newly suggested estimation method (blue) as function of different noise levels, created with a TASEP simulation with** ***high*** **initiation rates.**
(TIF)

Figure S6 **Estimation errors for the old (red) and the newly suggested estimation method (blue) as function of different noise levels, created with a TASEP simulation with** ***proportional*** **initiation rates.**
(TIF)

Figure S7 **Estimated** ***SL*** **points using both the old and the newly suggested methods on the ribosomal read counts profile of isoform uc007gge.1.**
(TIF)

Figure S8 **Explaining the semgents' length by using their tAI/CAI/folding energy/charge values.** Segments were divided into two groups (top/bottom 20%(black)/30%(red)/40%(blue)/50% (green)) according to their genes' (A.) tAI, (B.) CAI and segments' (C.) folding energy and (D.) charge values.
(TIF)

Figure S9 ***DSRC*** **measure calculated for simulated ribosomal profiles for low and high initiation rates; for each isoform we simulated 20 mRNAs.** (A.) Read count profiles created using a low initialization rate, constructed with all fragments or (B.) only fragments covered by monosomes. (C.) Read count profiles created using high initialization rate, constructed with all fragments or (D.) with fragments covered only by monosomes.
(TIF)

Figure S10 ***DSRC*** **measure calculated for simulated ribosomal profiles for low and high initiation rates; for each isoform we simulated 50 mRNAs.** (A.) Read count profiles created using a low initialization rate, constructed with all fragments or (B.) only fragments covered by monosomes. (C.) Read count profiles created using high initialization rate, constructed with all fragments or (D.) with fragments covered only by monosomes.
(TIF)

Figure S11 ***DSRC*** **measure calculated for simulated ribosomal profiles for low and high initiation rates; for each isoform we simulated 500 mRNAs.** (A.) Read count profiles created using a low initialization rate, constructed with all fragments or (B.) only fragments covered by monosomes. (C.) Read count profiles created using high initialization rate, constructed with all fragments or (D.) with fragments covered only by monosomes.
(TIF)

Figure S12
**Spearman correlation between** 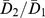 **and** 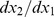 **calculated for simulated ribosomal profiles for low and high initiation rates; for each isoform we simulated 20 mRNAs.** (A.) Read count profiles created using a low initialization rate, constructed with all fragments or (B.) with fragments only covered by monosomes. (C.) Read count profiles created using high initialization rate, constructed with all fragments or (D.) with fragments covered only by monosomes.(TIF)

Figure S13
**Spearman correlation between** 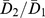 **and** 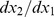 **calculated for simulated ribosomal profiles for low and high initiation rates; for each isoform we simulated 50 mRNAs.** (A.) Read count profiles created using a low initialization rate, constructed with all fragments or (B.) with fragments only covered by monosomes. (C.) Read count profiles created using high initialization rate, constructed with all fragments or (D.) with fragments covered only by monosomes.(TIF)

Figure S14
**Spearman correlation between** 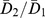 **and** 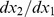 **calculated for simulated ribosomal profiles for low and high initiation rates; for each isoform we simulated 500 mRNAs.** (A.) Read count profiles created using a low initialization rate, constructed with all fragments or (B.) with fragments only covered by monosomes. (C.) Read count profiles created using high initialization rate, constructed with all fragments or (D.) with fragments covered only by monosomes.(TIF)

Figure S15
**Simulating the effect of unequal propagation time of harringtonine.** (A.) 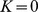 (B.) 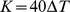 (C.) 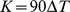 (D.) 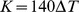. As can be seen from the results, an increased non-uniform harringtonine effect disturbs the profiles and decreases the slope of the run-off profiles.(TIF)

Figure S16
**Estimating the bias of** ***SL*** **points caused by non-uniform effect of harringtonine.** (A.) Mean and standard deviation of the *SL* points were calculated for each of the tested 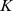 values, in comparison to the *SL* points calculated for 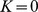. (B.) Velocities ratio 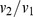 were calculated as function of the intensity of the non-uniform effect. As seen from the figure, for higher 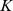 the bias of the estimated *SL* points increases; however, the ratio between the estimated velocities is almost not affected.(TIF)

Figure S17
**Calculating** ***DSRC*** **and the correlation between** 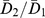 **and** 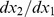 **under the effect of unequal propagation times of harringtonine.** As seen from the results, a higher non-uniform effect of harringtonine increases the *DSRC* measure and decreases the correlation between the 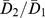 and the 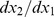 measures.(TIF)

Table S1 **Description of the analyzed data.**
(DOCX)

Table S2 **Alignment results.**
(DOCX)

Table S3 **Estimated** ***SL*** **locations using the old estimation method.** *SL* points locations were calculated for a recovery factor of 0.5 for profiles smoothed with averaging windows of different lengths (codon units).
(DOCX)

Table S4 **Estimated** ***SL*** **locations using the old estimation method.** *SL* points locations were calculated for different recovery factors for profiles smoothed with an averaging window of 30 codons.
(DOCX)

Table S5 **Estimated** ***SL*** **locations using the new estimation method.** *SL* points were calculated for a recovery factor of 0.5 for profiles smoothed with averaging windows of different lengths (codon units).
(DOCX)

Table S6 **Estimated** ***SL*** **locations using the new estimation method.** *SL* points were calculated for different recovery factors for profiles smoothed with an averaging window of 30 codons.
(DOCX)

Table S7 **Explaining the segments' length by using various features of the coding sequence.** Segments were divided into top/bottom 20%/30%/40%/50% according to their genes' tAI index/CAI index/segments' folding energy/segments' charge and were compared by using an unpaired t-test and two samples KS-test.
(DOCX)

Table S8
**DSRC values and Spearman correlation between** 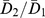 **and** 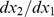 **for different recovery factors, when using both estimation methods.** Ribosomal densities were smoothed for all profiles using a window of 30 codons.(DOCX)

Table S9 **Flux ratios (mean and median values) for a recovery factor of 0.5, using both the old and new estimation methods. Ribosomal densities were smoothed for all profiles using a window of 5–30 codons.**
(DOCX)

Table S10 **Sheet 1: Isoforms in the analysis of this study were selected according to the criterion presented in the original work of Ingolia** ***et al.*** **, 2011.** Sheet 2: Calculated codons frequencies, based on ribosomal proteins (second column) and codons tAI value, calculation based on tRNA copy numbers (third column). Sheet 3: Selected ribosomal proteins for calculating the codon frequencies presented in the second sheet, second column.
(XLSX)

Text S1 **Supplementary methods.**
(DOCX)
