# Supplementary figures and images for: Determinants of Translation Elongation Speed and Ribosomal Profiling Biases in Mouse Embryonic Stem Cells
Source: PLoS Comput Biol. 2012 Nov 1;8(11):e1002755. doi: 10.1371/journal.pcbi.1002755 (PMC3486846; doi:10.1371/journal.pcbi.1002755)

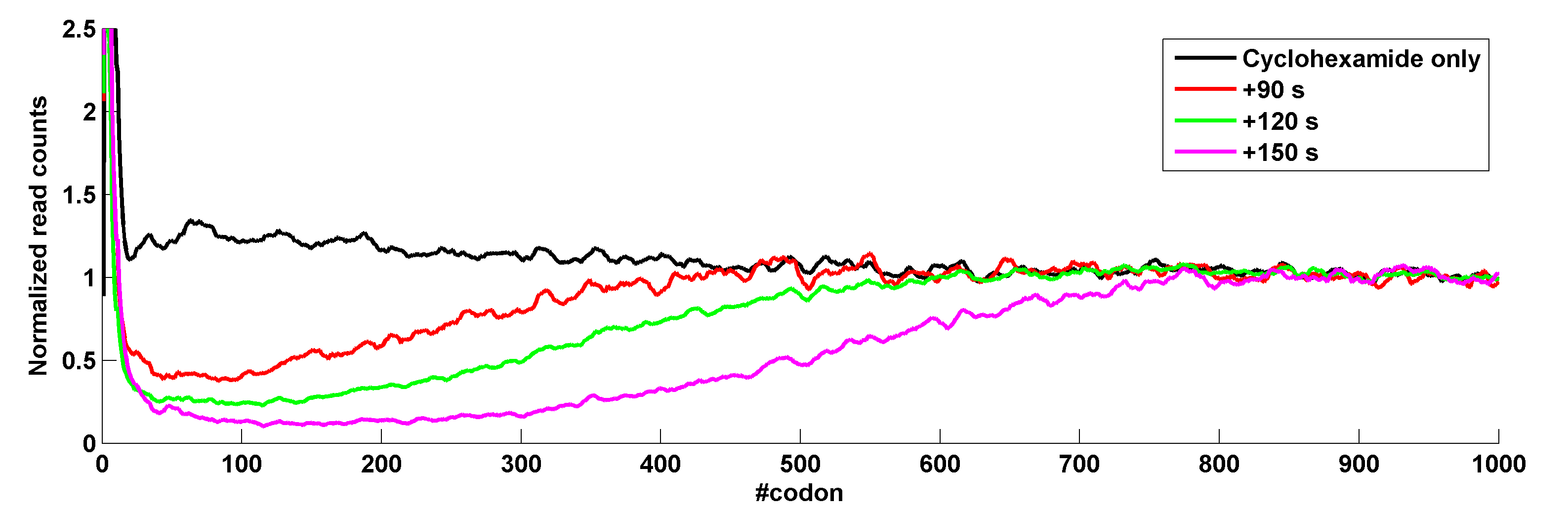

Supplement: Figure S1 — Reconstructed ribosomal profiles using real fragments, for different run-off times – average view. (TIF) [file pcbi.1002755.s001.tif]

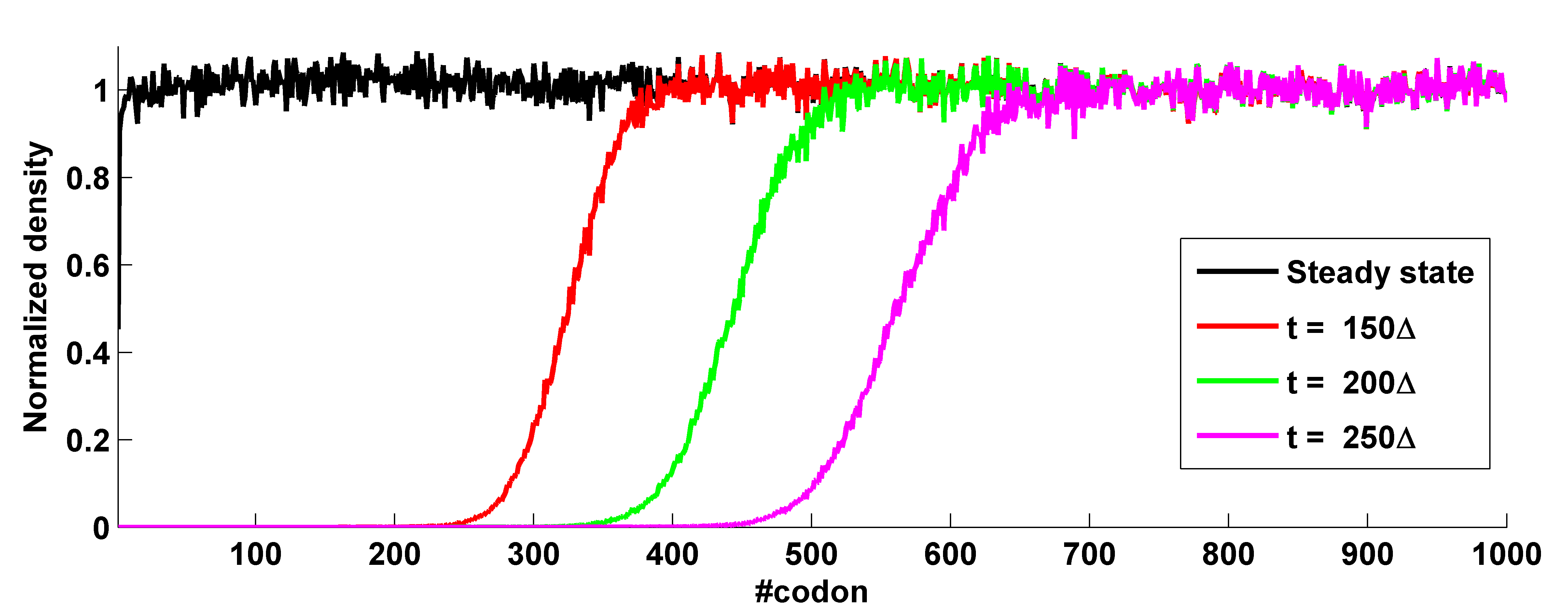

Supplement: Figure S2 — Simulated ribosomal profiles for different run-off times – average view. (TIF) [file pcbi.1002755.s002.tif]

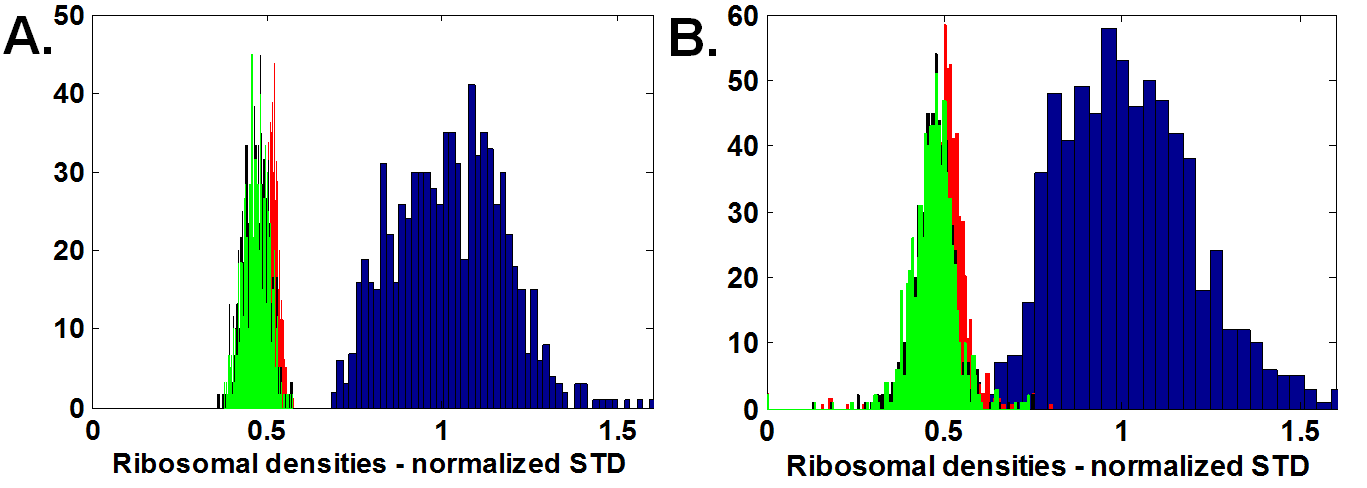

Supplement: Figure S3 — Histogram of the normalized standard deviation (STD) calculated for genes with good reads and with at least 1000 codons. The standard deviation was calculated using the real ribosomal profiles (blue) and based on simulative profiles created using the TASEP model. We considered different initiation rate regimes for the TASEP - low (red), high (black) and proportional (green). (A.) Normalized STD calculated on read counts of codons 730–1000. (B.) Normalized STD calculated on read counts of all codons, except for the first 40 and last 20 codons. (TIF) [file pcbi.1002755.s003.tif]

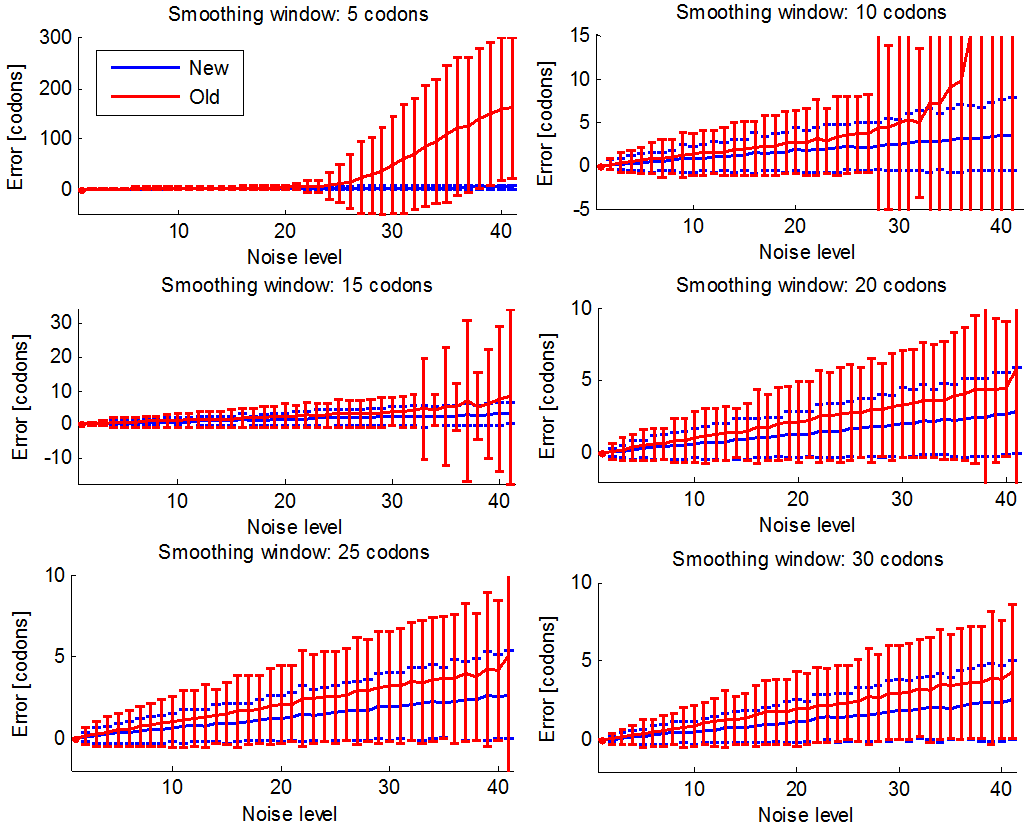

Supplement: Figure S4 — Estimation errors of the old (red) and the newly suggested estimation method (blue) as function of different noise levels, created with a TASEP simulation with low initiation rates. (TIF) [file pcbi.1002755.s004.tif]

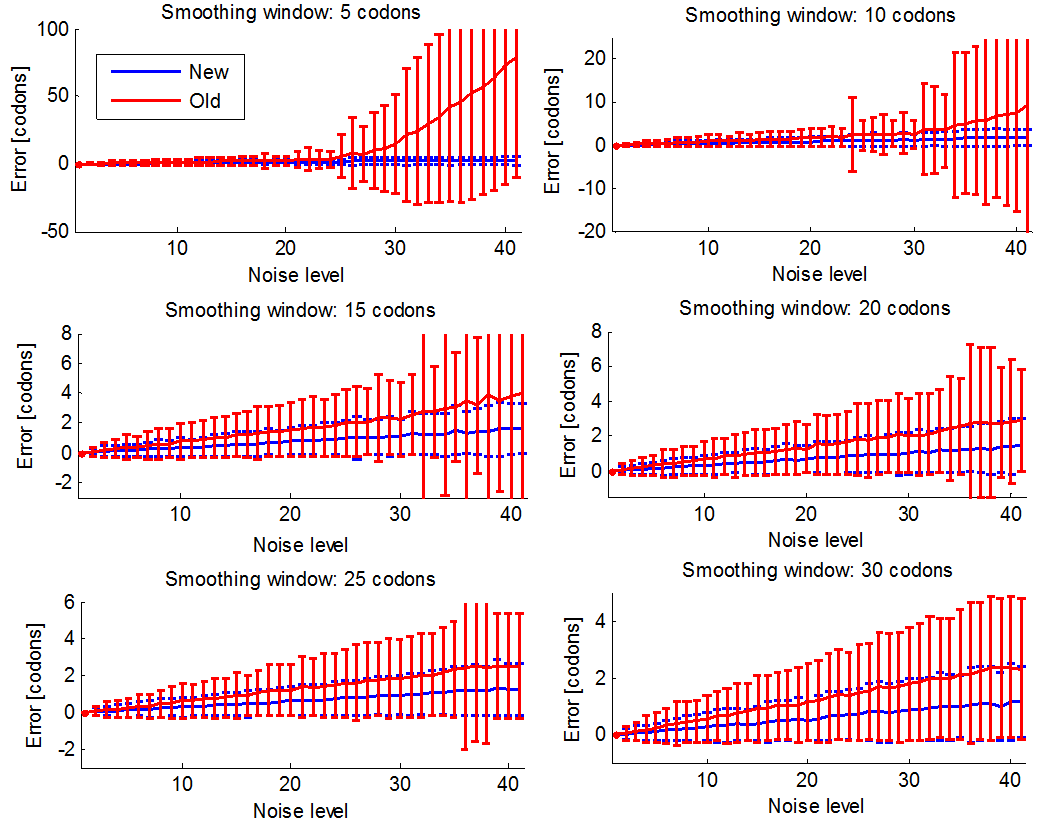

Supplement: Figure S5 — Estimation errors of the old (red) and the newly suggested estimation method (blue) as function of different noise levels, created with a TASEP simulation with high initiation rates. (TIF) [file pcbi.1002755.s005.tif]

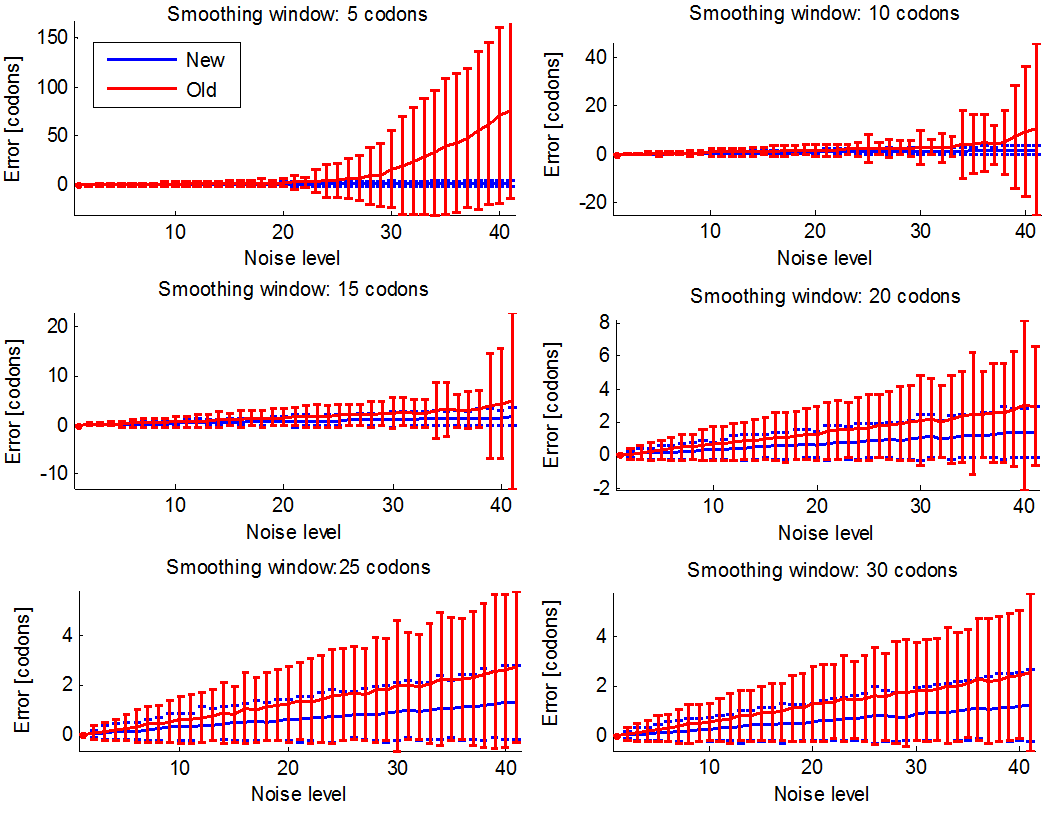

Supplement: Figure S6 — Estimation errors for the old (red) and the newly suggested estimation method (blue) as function of different noise levels, created with a TASEP simulation with proportional initiation rates. (TIF) [file pcbi.1002755.s006.tif]

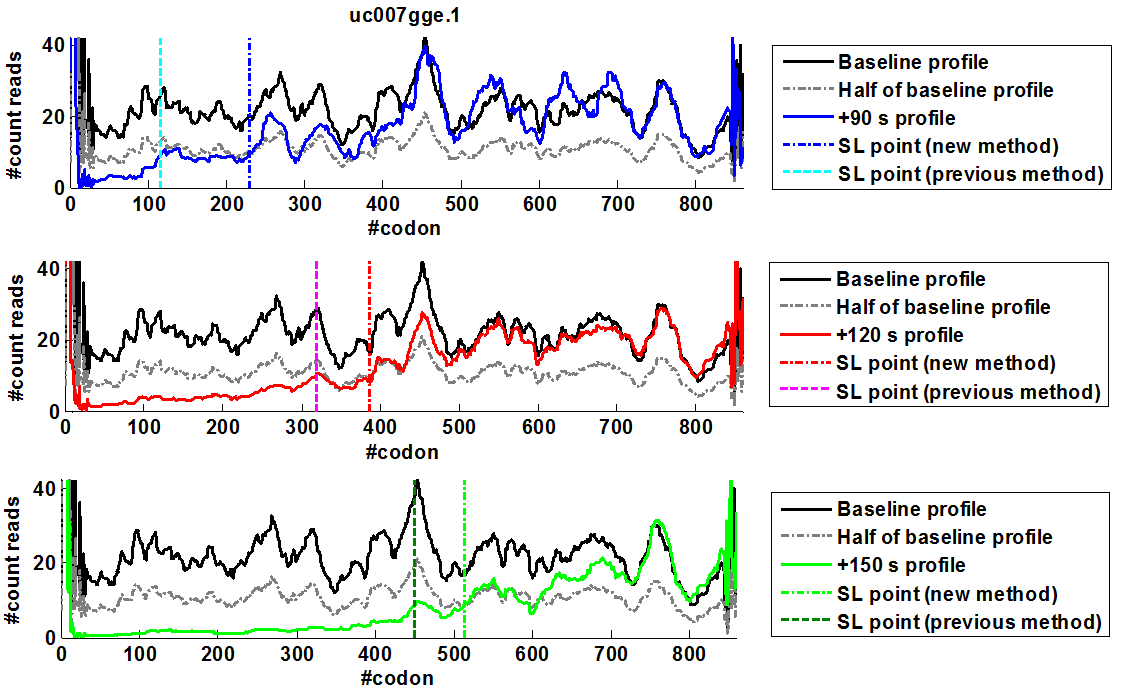

Supplement: Figure S7 — Estimated SL points using both the old and the newly suggested methods on the ribosomal read counts profile of isoform uc007gge.1. (TIF) [file pcbi.1002755.s007.tif]

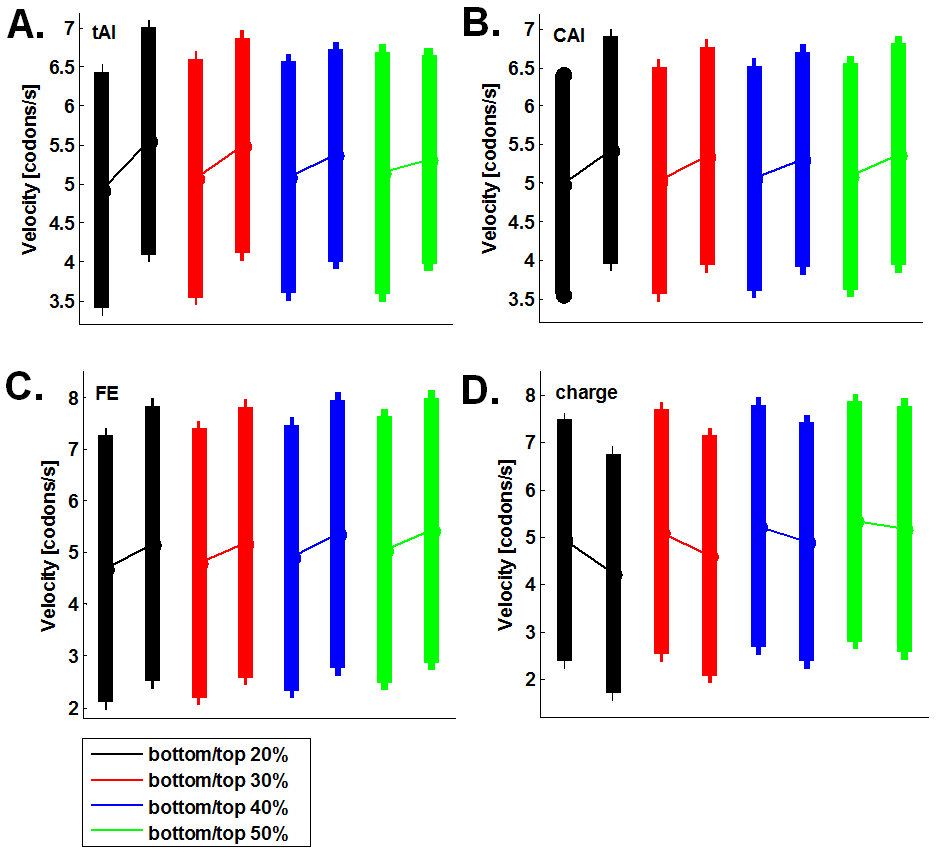

Supplement: Figure S8 — Explaining the semgents' length by using their tAI/CAI/folding energy/charge values. Segments were divided into two groups (top/bottom 20%(black)/30%(red)/40%(blue)/50% (green)) according to their genes' (A.) tAI, (B.) CAI and segments' (C.) folding energy and (D.) charge values. (TIF) [file pcbi.1002755.s008.tif]

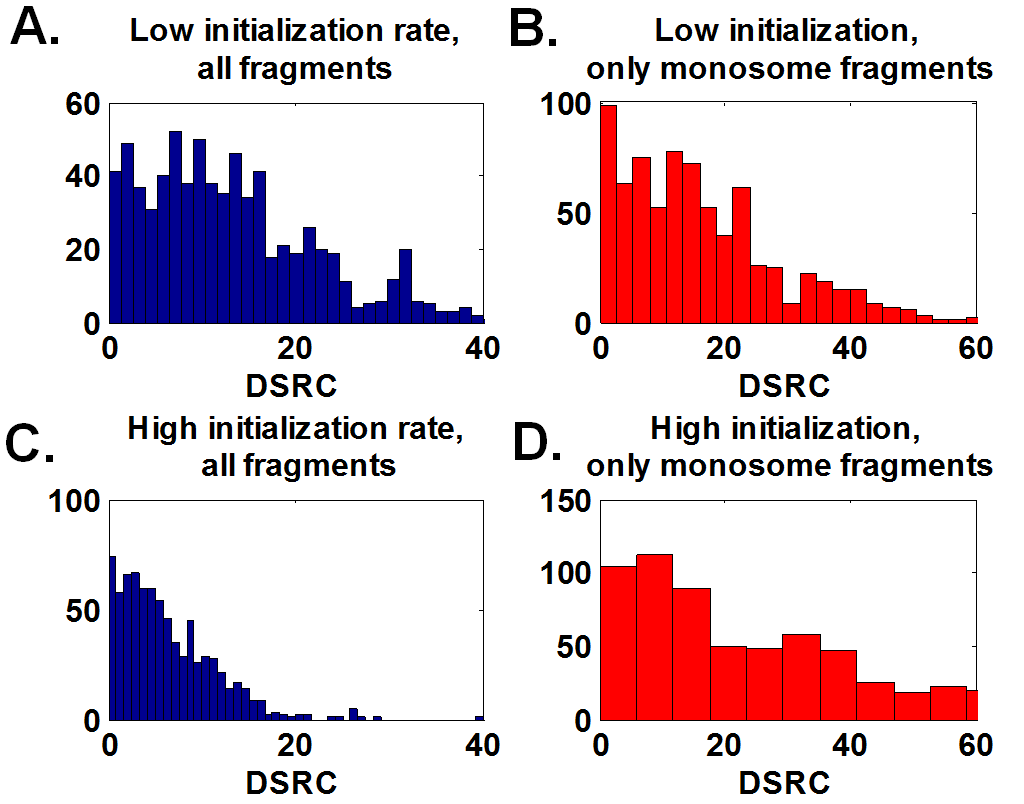

Supplement: Figure S9 — DSRC measure calculated for simulated ribosomal profiles for low and high initiation rates; for each isoform we simulated 20 mRNAs. (A.) Read count profiles created using a low initialization rate, constructed with all fragments or (B.) only fragments covered by monosomes. (C.) Read count profiles created using high initialization rate, constructed with all fragments or (D.) with fragments covered only by monosomes. (TIF) [file pcbi.1002755.s009.tif]

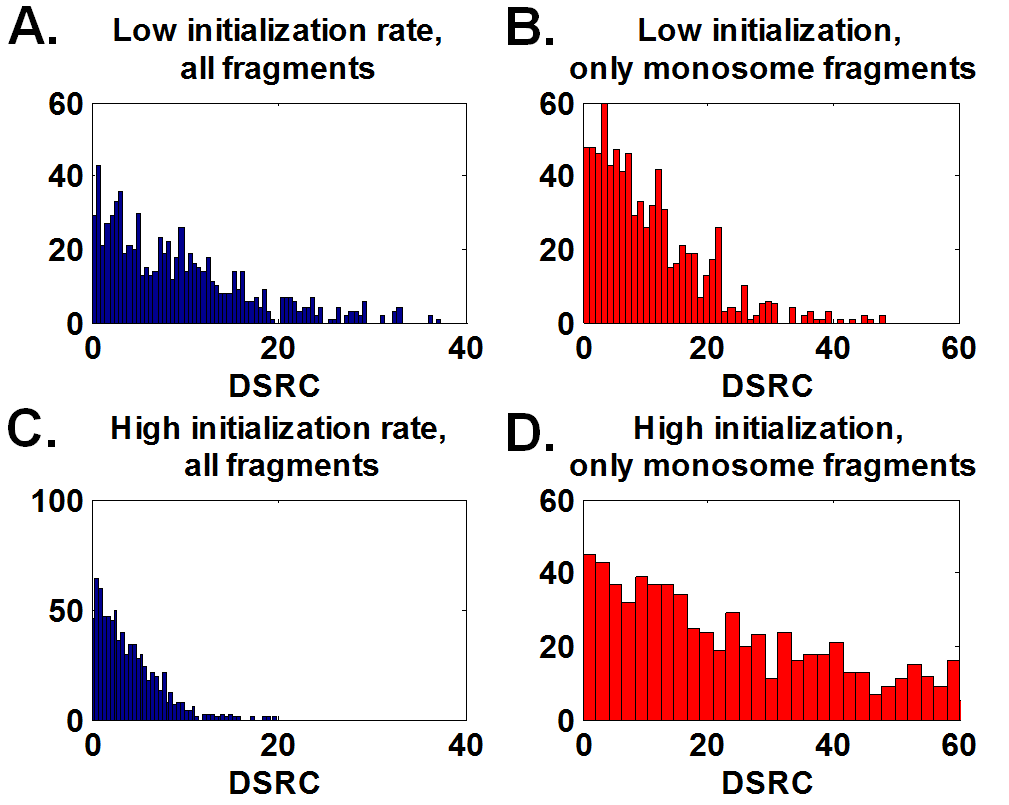

Supplement: Figure S10 — DSRC measure calculated for simulated ribosomal profiles for low and high initiation rates; for each isoform we simulated 50 mRNAs. (A.) Read count profiles created using a low initialization rate, constructed with all fragments or (B.) only fragments covered by monosomes. (C.) Read count profiles created using high initialization rate, constructed with all fragments or (D.) with fragments covered only by monosomes. (TIF) [file pcbi.1002755.s010.tif]

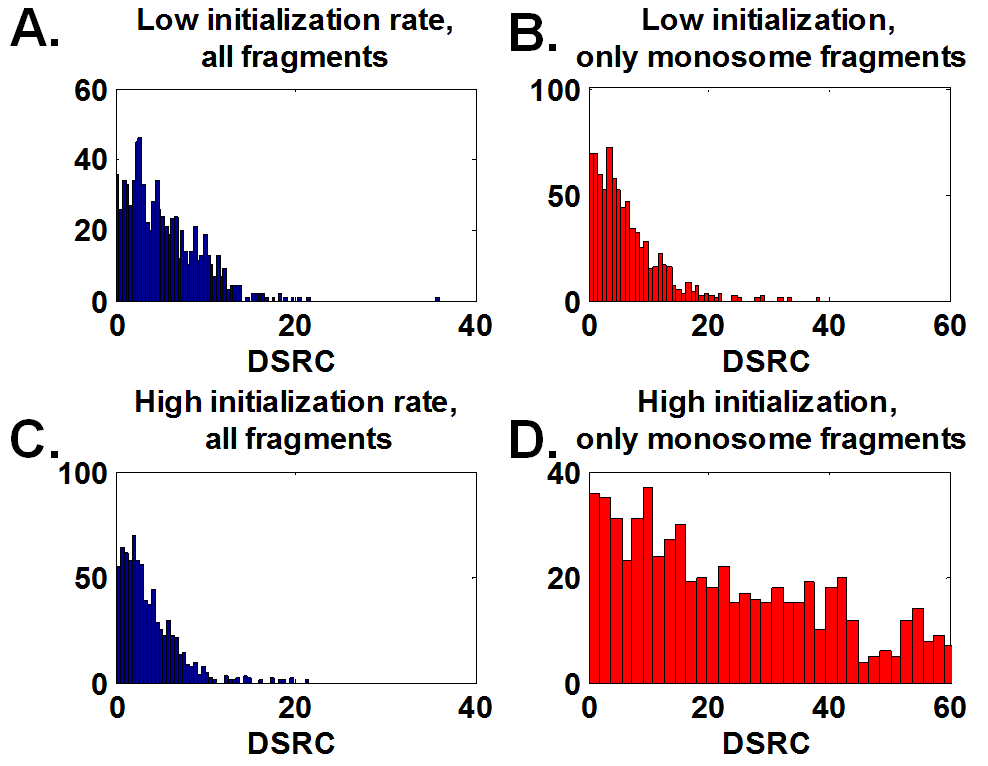

Supplement: Figure S11 — DSRC measure calculated for simulated ribosomal profiles for low and high initiation rates; for each isoform we simulated 500 mRNAs. (A.) Read count profiles created using a low initialization rate, constructed with all fragments or (B.) only fragments covered by monosomes. (C.) Read count profiles created using high initialization rate, constructed with all fragments or (D.) with fragments covered only by monosomes. (TIF) [file pcbi.1002755.s011.tif]

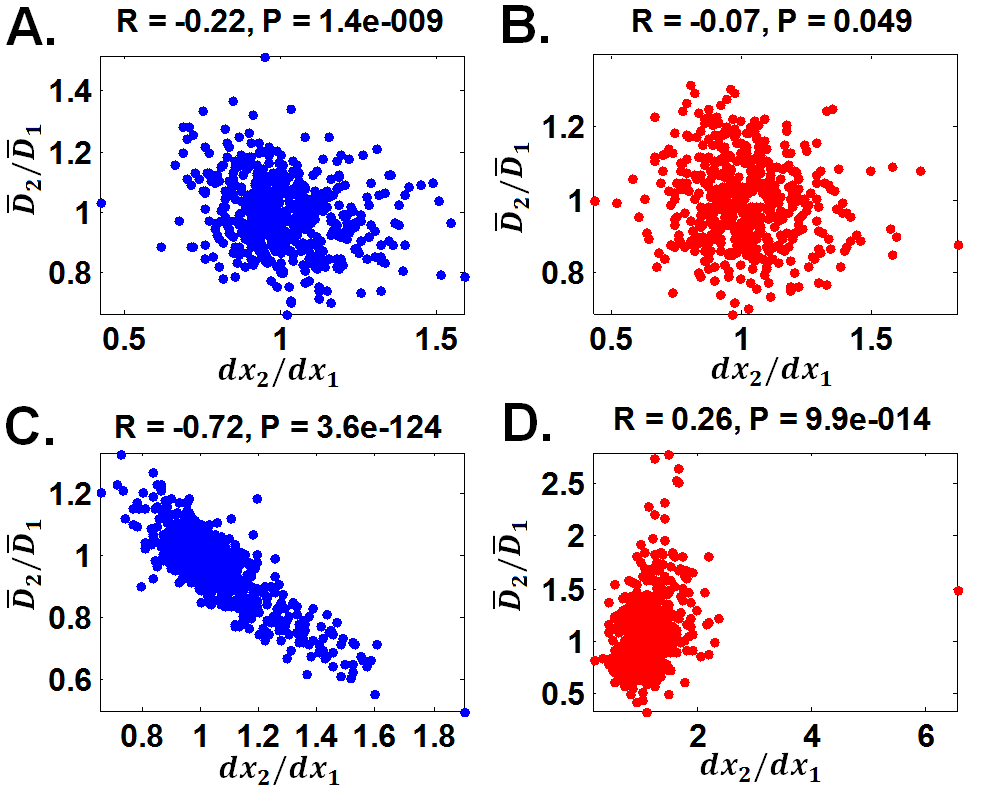

Supplement: Figure S12 — Spearman correlation between and calculated for simulated ribosomal profiles for low and high initiation rates; for each isoform we simulated 20 mRNAs. (A.) Read count profiles created using a low initialization rate, constructed with all fragments or (B.) with fragments only covered by monosomes. (C.) Read count profiles created using high initialization rate, constructed with all fragments or (D.) with fragments covered only by monosomes. (TIF) [file pcbi.1002755.s012.tif]

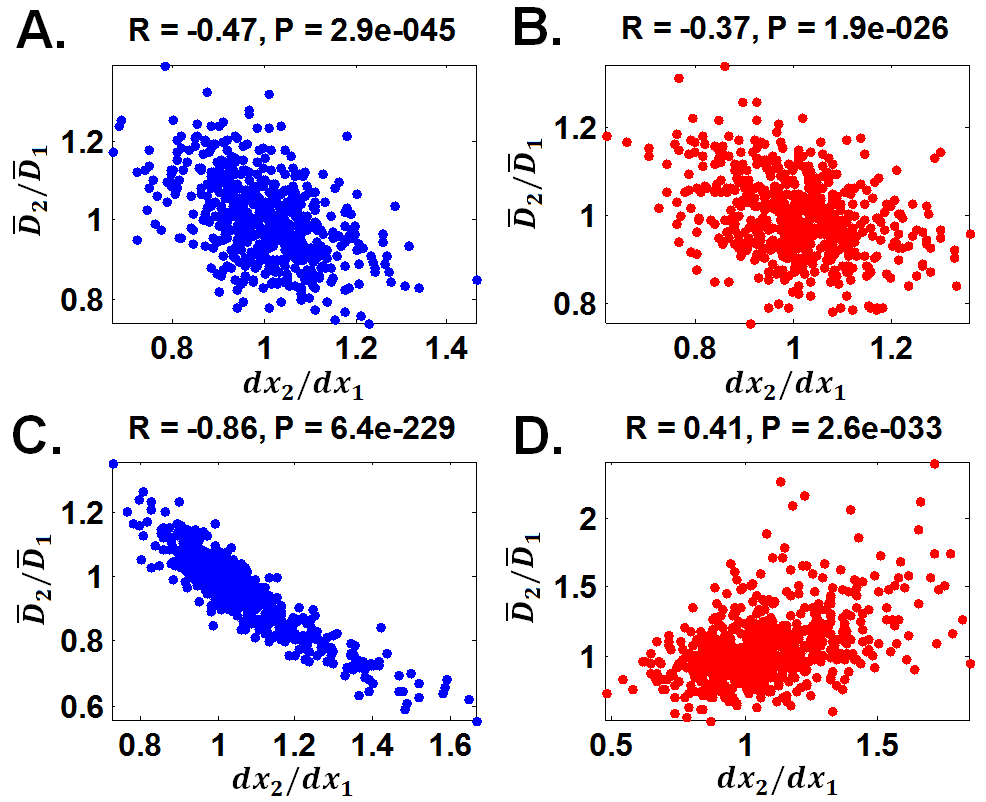

Supplement: Figure S13 — Spearman correlation between and calculated for simulated ribosomal profiles for low and high initiation rates; for each isoform we simulated 50 mRNAs. (A.) Read count profiles created using a low initialization rate, constructed with all fragments or (B.) with fragments only covered by monosomes. (C.) Read count profiles created using high initialization rate, constructed with all fragments or (D.) with fragments covered only by monosomes. (TIF) [file pcbi.1002755.s013.tif]

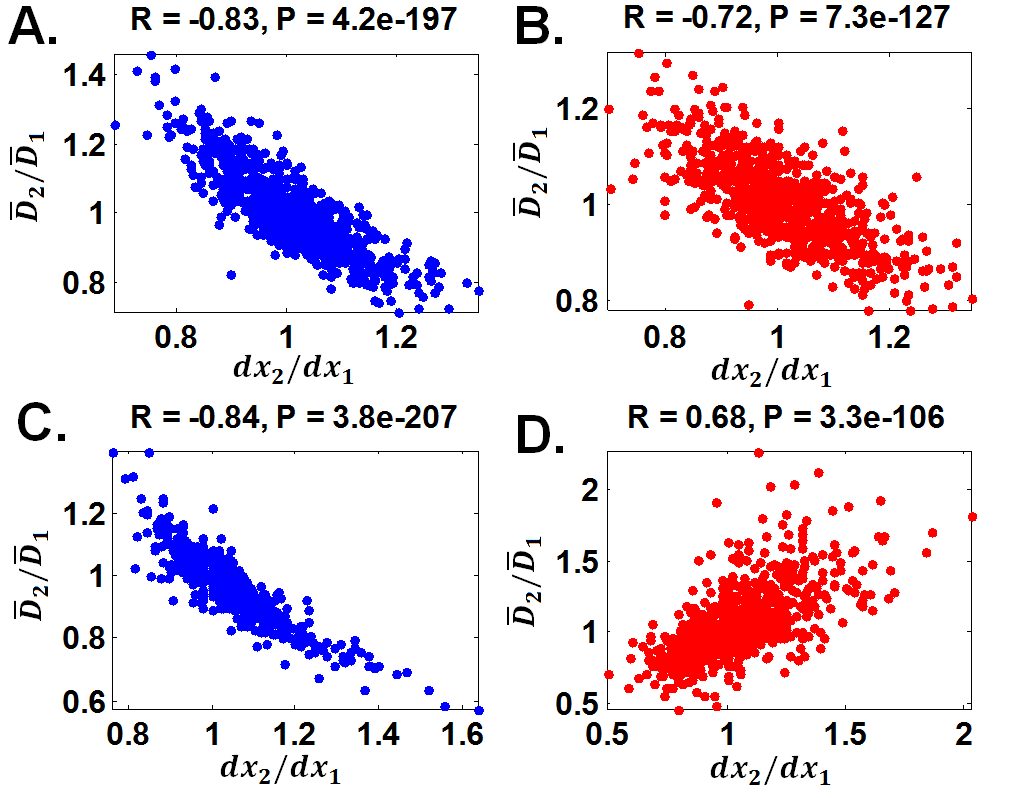

Supplement: Figure S14 — Spearman correlation between and calculated for simulated ribosomal profiles for low and high initiation rates; for each isoform we simulated 500 mRNAs. (A.) Read count profiles created using a low initialization rate, constructed with all fragments or (B.) with fragments only covered by monosomes. (C.) Read count profiles created using high initialization rate, constructed with all fragments or (D.) with fragments covered only by monosomes. (TIF) [file pcbi.1002755.s014.tif]

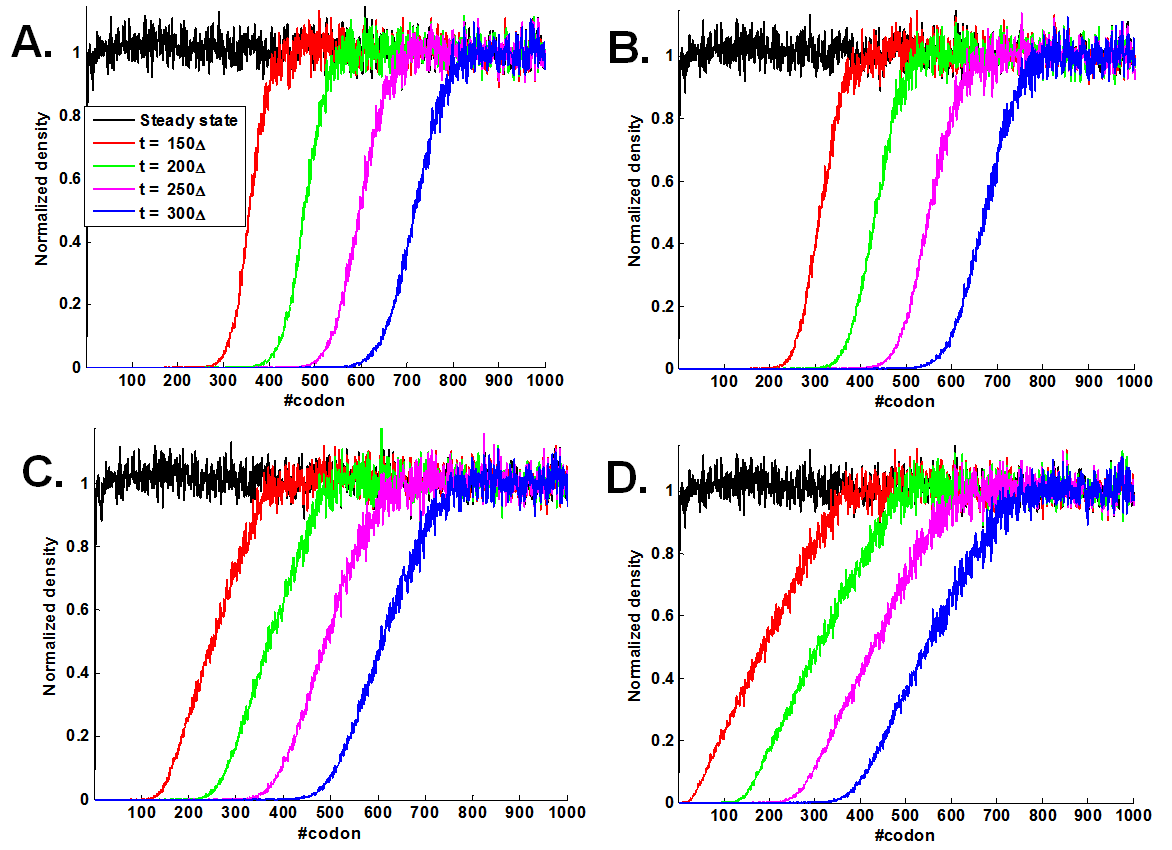

Supplement: Figure S15 — Simulating the effect of unequal propagation time of harringtonine. (A.) (B.) (C.) (D.) . As can be seen from the results, an increased non-uniform harringtonine effect disturbs the profiles and decreases the slope of the run-off profiles. (TIF) [file pcbi.1002755.s015.tif]

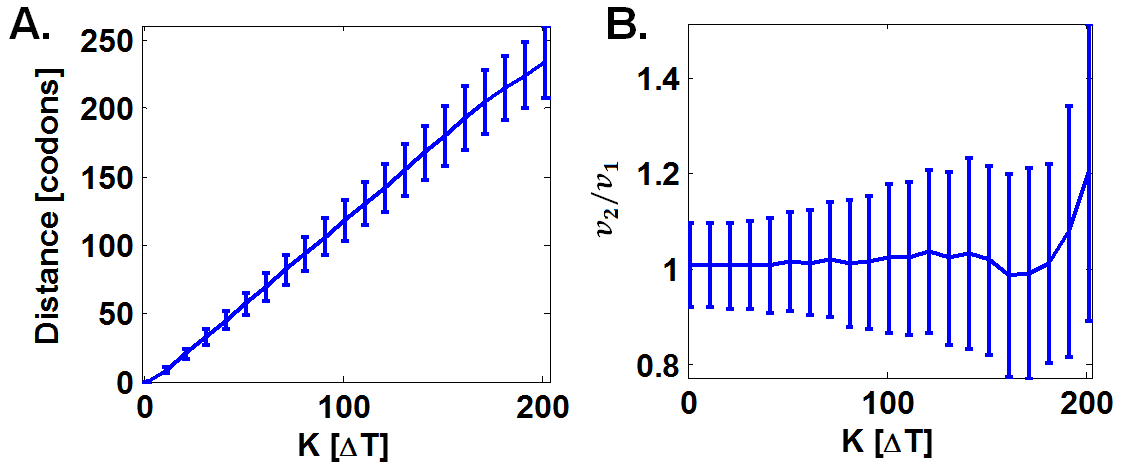

Supplement: Figure S16 — Estimating the bias of SL points caused by non-uniform effect of harringtonine. (A.) Mean and standard deviation of the SL points were calculated for each of the tested values, in comparison to the SL points calculated for . (B.) Velocities ratio were calculated as function of the intensity of the non-uniform effect. As seen from the figure, for higher the bias of the estimated SL points increases; however, the ratio between the estimated velocities is almost not affected. (TIF) [file pcbi.1002755.s016.tif]

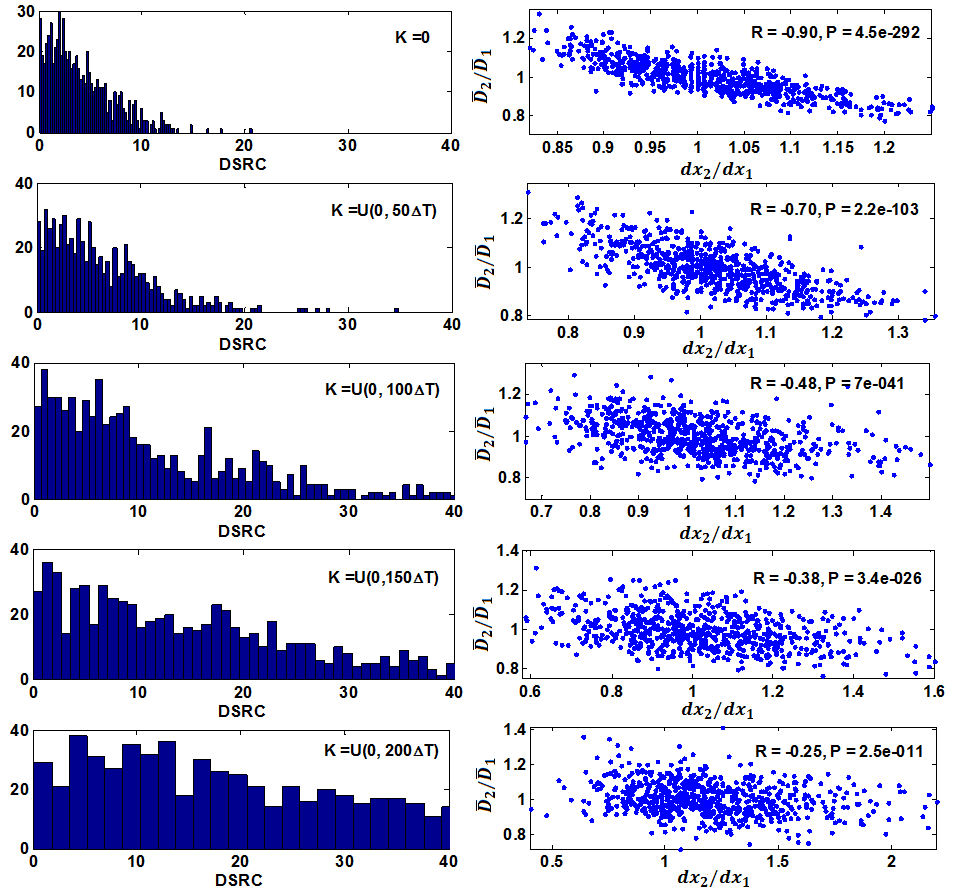

Supplement: Figure S17 — Calculating DSRC and the correlation between and under the effect of unequal propagation times of harringtonine. As seen from the results, a higher non-uniform effect of harringtonine increases the DSRC measure and decreases the correlation between the and the measures. (TIF) [file pcbi.1002755.s017.tif]
