# Supplementary material for: Determinants of Translation Elongation Speed and Ribosomal Profiling Biases in Mouse Embryonic Stem Cells
Source: PLoS Comput Biol. 2012 Nov 1;8(11):e1002755. doi: 10.1371/journal.pcbi.1002755 (PMC3486846; doi:10.1371/journal.pcbi.1002755)
Supplement: Table S1 — Description of the analyzed data. (DOCX) [file pcbi.1002755.s018.docx]

| **Description** | **Sample ID** | **Internal name** | **Source** |
| --- | --- | --- | --- |
| Protected RNA fragments  Cyclohexamide treatment | GSM765292 | CYH60(1) | <ftp://ftp.ncbi.nih.gov/pub/geo/DATA/supplementary/samples/GSM765nnn/GSM765292/GSM765292_ribo_mesc_chx_100426l5_trxs_norrna.align.txt.gz> |
|  |  | CYH60(2) | <ftp://ftp.ncbi.nih.gov/pub/geo/DATA/supplementary/samples/GSM765nnn/GSM765292/GSM765292_ribo_mesc_chx_circsub_100809l4_trxs_norrna.align.txt.gz> |
| Protected RNA fragments  Harigntonine +  Cyclohexamide applied after 90 s | GSM765297 | CYH90(1) | <ftp://ftp.ncbi.nih.gov/pub/geo/DATA/supplementary/samples/GSM765nnn/GSM765297/GSM765297_ribo_mesc_harr90s_100527l1_trxs_norrna.align.txt.gz> |
|  |  | CYH90(2) | <ftp://ftp.ncbi.nih.gov/pub/geo/DATA/supplementary/samples/GSM765nnn/GSM765297/GSM765297_ribo_mesc_harr90s_circsub_100624l1_trxs_norrna.align.txt.gz> |
|  |  | CYH90(3) | <ftp://ftp.ncbi.nih.gov/pub/geo/DATA/supplementary/samples/GSM765nnn/GSM765297/GSM765297_ribo_mesc_harr90s_circsub_100706l1_trxs_norrna.align.txt.gz> |
| Protected RNA fragments  Harigntonine +  Cyclohexamide applied after 120 s | GSM765294 | CYH120(1) | <ftp://ftp.ncbi.nih.gov/pub/geo/DATA/supplementary/samples/GSM765nnn/GSM765294/GSM765294_ribo_mesc_harr120s_100527l2_trxs_norrna.align.txt.gz> |
|  |  | CYH120(2) | <ftp://ftp.ncbi.nih.gov/pub/geo/DATA/supplementary/samples/GSM765nnn/GSM765294/GSM765294_ribo_mesc_harr120s_circsub_100706l5_trxs_norrna.align.txt.gz> |
|  |  | CYH120(3) | <ftp://ftp.ncbi.nih.gov/pub/geo/DATA/supplementary/samples/GSM765nnn/GSM765294/GSM765294_ribo_mesc_harr120s_circsub_100706l6_trxs_norrna.align.txt.gz> |
| Protected RNA fragments  Harigntonine +  Cyclohexamide applied after 150 s | GSM765295 | CYH150(1) | <ftp://ftp.ncbi.nih.gov/pub/geo/DATA/supplementary/samples/GSM765nnn/GSM765295/GSM765295_ribo_mesc_harr150s_100527l3_trxs_norrna.align.txt.gz> |
|  |  | CYH150(2) | <ftp://ftp.ncbi.nih.gov/pub/geo/DATA/supplementary/samples/GSM765nnn/GSM765295/GSM765295_ribo_mesc_harr150s_circsub_100624l2_trxs_norrna.align.txt.gz> |
|  |  | CYH150(3) | <ftp://ftp.ncbi.nih.gov/pub/geo/DATA/supplementary/samples/GSM765nnn/GSM765295/GSM765295_ribo_mesc_harr150s_circsub_100706l2_trxs_norrna.align.txt.gz> |
