# Supplementary material for: Determinants of Translation Elongation Speed and Ribosomal Profiling Biases in Mouse Embryonic Stem Cells
Source: PLoS Comput Biol. 2012 Nov 1;8(11):e1002755. doi: 10.1371/journal.pcbi.1002755 (PMC3486846; doi:10.1371/journal.pcbi.1002755)
Supplement: Table S2 — Alignment results. (DOCX) [file pcbi.1002755.s019.docx]

| **File** | **#Fragments** | **% processed** | **%aligned** | **% to 1 loc** | **% to 2 locs** | **% to 3 locs** | **% > 3 locs** |
| --- | --- | --- | --- | --- | --- | --- | --- |
| **CYH60(1)** | 5714909 | 96.2 | 73.73 | 78.2 | 15.2 | 1.7 | 4.8 |
| **CYH60(2)** | 11463830 | 97.92 | 91.8 | 78.2 | 15.3 | 1.7 | 4.7 |
| **CYH90(1)** | 2878682 | 94.35 | 92.35 | 82 | 13.6 | 1.5 | 2.8 |
| **CYH90(2)** | 7953773 | 98.8 | 93.52 | 81.5 | 14.2 | 1.5 | 2.9 |
| **CYH90(3)** | 6633489 | 98.77 | 93.4 | 81.4 | 14.2 | 1.5 | 2.9 |
| **CYH120(1)** | 4597983 | 95.09 | 92.05 | 83.9 | 12.8 | 1.3 | 2.1 |
| **CYH120(2)** | 7807891 | 97.05 | 92.34 | 83.8 | 12.9 | 1.3 | 2 |
| **CYH120(3)** | 7846937 | 97.05 | 92.34 | 83.7 | 12.9 | 1.3 | 2.1 |
| **CYH150(1)** | 3226273 | 93.77 | 90.96 | 84.4 | 12 | 1.4 | 2.1 |
| **CYH150(2)** | 7347546 | 96.07 | 91.36 | 84.4 | 12.1 | 1.4 | 2.1 |
| **CYH150(3)** | 6132213 | 95.95 | 91.23 | 84.2 | 12.2 | 1.4 | 2.2 |
