# Supplementary material for: Determinants of Translation Elongation Speed and Ribosomal Profiling Biases in Mouse Embryonic Stem Cells
Source: PLoS Comput Biol. 2012 Nov 1;8(11):e1002755. doi: 10.1371/journal.pcbi.1002755 (PMC3486846; doi:10.1371/journal.pcbi.1002755)
Supplement: Table S3 — Estimated SL locations using the old estimation method. SL points locations were calculated for a recovery factor of 0.5 for profiles smoothed with averaging windows of different lengths (codon units). (DOCX) [file pcbi.1002755.s020.docx]

| Smoothing window size | $x_{1}$ [codons] | $x_{2}$ [codons] | $x_{3}$  [codons] | mean($v_{1}$) [codons/  second] | mean($v_{2}$) [codons/  second] | KS-test P value | Mean($v_{1}$,$v_{2}$) [codons/second] | Median of $v_{2}$/$v_{1}$ [codons/second] | Median of  \|$v_{1}-v2\vert/min(v_{1},v_{2})$ | Number of genes $x_{1}{<x}_{2}{<x}_{3}$ |
| --- | --- | --- | --- | --- | --- | --- | --- | --- | --- | --- |
| 5 | 74+/-21 | 141+/-70 | 238+/-113 | 2.2+/-2.2 | 2.7+/-0.7 | <2.25*10^-6^ | 2.9+/-0.8 | 1.69 | 1.83 | 375 |
| 10 | 87+/-37 | 198+/-83 | 334+/-115 | 3.7+/-2.5 | 4.5+/-2.9 | <5.63*10^-4^ | 4.1+/-0.6 | 1.15 | 1.12 | 525 |
| 15 | 94+/-41 | 227+/-83 | 371+/-112 | 4.4+/-2.5 | 4.8+/-2.7 | <0.085 | 4.6+/-0.3 | 1.04 | 0.81 | 569 |
| 20 | 105+/-50 | 249+/-82 | 408+/-101 | 4.8+/-2.5 | 5.3+/-2.6 | <0.0541 | 5.1+/-0.3 | 1.08 | 0.76 | 595 |
| 25 | 110+/-54 | 258+/-80 | 422+/-97 | 4.9+/-2.4 | 5.5+/-2.5 | <2.35*10^-4^ | 5.2+/-0.4 | 1.09 | 0.74 | 614 |
| 30 | 116+/-57 | 265+/-82 | 433+/-102 | 5.0+/-2.5 | 5.6+/-2.7 | <1.74*10^-5^ | 5.3+/-0.4 | 1.11 | 0.75 | 644 |
