# Supplementary material for: Determinants of Translation Elongation Speed and Ribosomal Profiling Biases in Mouse Embryonic Stem Cells
Source: PLoS Comput Biol. 2012 Nov 1;8(11):e1002755. doi: 10.1371/journal.pcbi.1002755 (PMC3486846; doi:10.1371/journal.pcbi.1002755)
Supplement: Table S4 — Estimated SL locations using the old estimation method. SL points locations were calculated for different recovery factors for profiles smoothed with an averaging window of 30 codons. (DOCX) [file pcbi.1002755.s021.docx]

| Recovery factor | $x_{1}$ [codons] | $x_{2}$ [codons] | $x_{3}$  [codons] | mean($v_{1}$) [codons/  second] | mean($v_{2}$) [codons/  second] | KS-test P value | Mean($v_{1}$,$v_{2}$) [codons/second] | Median of $v_{2}$/$v_{1}$ [codons/second] | Median of  \|$v_{1}-v2\vert/min(v_{1},v_{2})$ | Number of genes $x_{1}{<x}_{2}{<x}_{3}$ |
| --- | --- | --- | --- | --- | --- | --- | --- | --- | --- | --- |
| 0.4 | 97+/-45 | 229+/-77 | 395+/-98 | 4.4+/-2.3 | 5.5+/-2.6 | <2.99*10^-14^ | 5.0+/-0.8 | 1.23 | 0.72 | 591 |
| 0.5 | 116+/-57 | 265+/-82 | 433+/-102 | 5.0+/-2.5 | 5.6+/-2.7 | <1.74*10^-5^ | 5.3+/-0.4 | 1.11 | 0.75 | 644 |
| 0.6 | 136+/-69 | 300+/-83 | 478+/-99 | 5.5+/-2.6 | 5.7+/-2.7 | <0.51 | 5.6+/-0.1 | 0.98 | 0.69 | 676 |
| 0.7 | 156+/-77 | 338+/-79 | 504+/-94 | 6.1+/-2.7 | 5.5+/-2.6 | <0.0027 | 5.8+/-0.4 | 0.92 | 0.71 | 690 |
| 0.8 | 180+/-88 | 371+/-86 | 534+/-99 | 6.4+/-2.9 | 5.4+/-2.7 | <1.06*10^-10^ | 5.9+/-0.7 | 0.90 | 0.76 | 691 |
