# Supplementary material for: Determinants of Translation Elongation Speed and Ribosomal Profiling Biases in Mouse Embryonic Stem Cells
Source: PLoS Comput Biol. 2012 Nov 1;8(11):e1002755. doi: 10.1371/journal.pcbi.1002755 (PMC3486846; doi:10.1371/journal.pcbi.1002755)
Supplement: Table S5 — Estimated SL locations using the new estimation method. SL points were calculated for a recovery factor of 0.5 for profiles smoothed with averaging windows of different lengths (codon units). (DOCX) [file pcbi.1002755.s022.docx]

| Smoothing window size | $x_{1}$ [codons] | $x_{2}$ [codons] | $x_{3}$  [codons] | mean($v_{1}$) [codons/  second] | mean($v_{2}$) [codons/  second] | KS-test P value | Mean($v_{1}$,$v_{2}$) [codons/second] | Median of $v_{2}$/$v_{1}$ [codons/second] | Median of  \|$v_{1}-v2\vert/min(v_{1},v_{2})$ | Number of genes $x_{1}{<x}_{2}{<x}_{3}$ |
| --- | --- | --- | --- | --- | --- | --- | --- | --- | --- | --- |
| 5 | 206+/-76 | 305+/-68 | 509+/-85 | 3.3+/-2.3 | 6.8+/-2.6 | <6.52*10^-74^ | 5.0+/-2.5 | 2.21 | 1.44 | 533 |
| 10 | 182+/-74 | 299+/-70 | 489+/-84 | 4.1+/-2.5 | 6.2+/-2.5 | <4.27*10^-42^ | 5.1+/-1.5 | 1.91 | 1.78 | 650 |
| 15 | 178+/-74 | 301+/-73 | 486+/-87 | 4.1+/-2.7 | 6.6+/-2.7 | <6.9*10^-35^ | 5.4+/-1.8 | 1.53 | 0.93 | 670 |
| 20 | 177+/-73 | 303+/-73 | 487+/-86 | 4.2+/-2.5 | 6.1+/-2.5 | <6.06*10^-30^ | 5.2+/-1.3 | 1.49 | 0.94 | 683 |
| 25 | 176+/-74 | 304+/-7 | 485+/-87 | 4.3+/-2.5 | 6.0+/-2.5 | <3.44*10^-28^ | 5.1+/-1.2 | 1.43 | 0.9 | 686 |
| 30 | ‎177+/-74 | ‎308+/-72 | ‎489+/-85 | ‎4.3+/-2.6 | ‎6.0+/-2.5 | ‎<1.78*10^-24^ | 5.2+/-1.2 | ‎1.37 | 0.82 | ‎692‎ |
