# Supplementary material for: Determinants of Translation Elongation Speed and Ribosomal Profiling Biases in Mouse Embryonic Stem Cells
Source: PLoS Comput Biol. 2012 Nov 1;8(11):e1002755. doi: 10.1371/journal.pcbi.1002755 (PMC3486846; doi:10.1371/journal.pcbi.1002755)
Supplement: Table S6 — Estimated SL locations using the new estimation method. SL points were calculated for different recovery factors for profiles smoothed with an averaging window of 30 codons. (DOCX) [file pcbi.1002755.s023.docx]

| Recovery factor | $x_{1}$ [codons] | $x_{2}$ [codons] | $x_{3}$  [codons] | mean($v_{1}$) [codons/  second] | mean($v_{2}$) [codons/  second] | KS-test P value | Mean($v_{1}$,$v_{2}$) [codons/second] | Median of $v_{2}$/$v_{1}$ [codons/second] | Median of  \|$v_{1}-v2\vert/min(v_{1},v_{2})$ | Number of genes $x_{1}{<x}_{2}{<x}_{3}$ |
| --- | --- | --- | --- | --- | --- | --- | --- | --- | --- | --- |
| 0.4 | 142+/-64 | 267+/-65 | 445+/-87 | 4.1+/-2.2 | 5.9+/-2.4 | ‎<1.85*10^-36^ | 5.0+/-1.3 | 1.45 | 0.81 | 692 |
| 0.5 | ‎177+/-74 | ‎308+/-72 | ‎489+/-85 | ‎4.3+/-2.6 | ‎6.0+/-2.5 | ‎<1.78*10^-24^ | 5.2+/-1.2 | ‎1.37 | 0.82 | ‎692‎ |
| 0.6 | 207+/-81 | 347+/-72 | 525+/-88 | 4.7+/-2.7 | 5.9+/-2.6 | ‎<5.96*10^-15^ | 5.3+/-0.9 | 1.24 | 0.81 | 691 |
| 0.7 | 237+/-88 | 389+/-77 | 561+/-93 | 5.0+/-2.9 | 5.7+/-2.7 | ‎<2.52*10^-07^ | 5.4+/-0.5 | 1.14 | 0.86 | 677 |
| 0.8 | 270+/-96 | 431+/-84 | 598+/-92 | 5.4+/-3.2 | 5.6+/-2.8 | ‎<0.0296 | 5.5+/-0.1 | 1.06 | 0.91 | 651 |
