# Supplementary material for: Determinants of Translation Elongation Speed and Ribosomal Profiling Biases in Mouse Embryonic Stem Cells
Source: PLoS Comput Biol. 2012 Nov 1;8(11):e1002755. doi: 10.1371/journal.pcbi.1002755 (PMC3486846; doi:10.1371/journal.pcbi.1002755)
Supplement: Table S7 — Explaining the segments' length by using various features of the coding sequence. Segments were divided into top/bottom 20%/30%/40%/50% according to their genes' tAI index/CAI index/segments' folding energy/segments' charge and were compared by using an unpaired t-test and two samples KS-test. (DOCX) [file pcbi.1002755.s024.docx]

| Top/bottom | tAI | | CAI | | Folding energy | | Charge | |
| --- | --- | --- | --- | --- | --- | --- | --- | --- |
|  | t-test | ks-test | t-test | ks-test | t-test | ks-test | t-test | ks-test |
| 20% | 0.00049 | 0.081 | ‎0.014 | 0.14 | 0.0036 | 0.04 | 0.023 | 0.0019 |
| 30% | 0.00039 | 0.045 | ‎0.028 | 0.5 | 0.033 | 0.0099 | 0.02 | 0.013 |
| 40% | ‎0.023 | 0.21 | ‎0.049 | 0.54 | 0.0049 | 0.0018 | 0.083 | 0.023 |
| 50% | ‎0.14 | 0.25 | ‎0.011 | 0.37 | 0.0073 | 0.0068 | 0.12 | 0.15 |
