# Supplementary material for: Determinants of Translation Elongation Speed and Ribosomal Profiling Biases in Mouse Embryonic Stem Cells
Source: PLoS Comput Biol. 2012 Nov 1;8(11):e1002755. doi: 10.1371/journal.pcbi.1002755 (PMC3486846; doi:10.1371/journal.pcbi.1002755)
Supplement: Table S8 — DSRC values and Spearman correlation between and for different recovery factors, when using both estimation methods. Ribosomal densities were smoothed for all profiles using a window of 30 codons. (DOCX) [file pcbi.1002755.s025.docx]

| Depletion recovery factor | Old method | | New method | |
| --- | --- | --- | --- | --- |
|  | median(DSRC) | $\bar{D}_{2}/\bar{D}_{1}$ to $dx_{2}/dx_{1}$ correlation | median(DSRC) | $\bar{D}_{2}/\bar{D}_{1}$ to $dx_{2}/dx_{1}$ correlation |
| 0.4 | 69 | R = -0.19, P < 10^-6^ | 77 | R = 0.08, P < 0.003 |
| 0.5 | 73 | R = -0.08, P < 2*10^-3^ | 88 | R = 0.13, P < 9*10^-4^ |
| 0.6 | 71 | R = -0.09, P <6*10^-6^ | 78 | R = 0.13, P = 6*10^-5^ |
| 0.7 | 67 | R = -0.06, P < 4*10^-4^ | 87 | R = 0.16, P = 4*10^-5^ |
| 0.8 | 78 | R = 0, P < 0.042 | 91 | R = 0.19, P = 6*10^-7^ |
