# Supplementary material for: Determinants of Translation Elongation Speed and Ribosomal Profiling Biases in Mouse Embryonic Stem Cells
Source: PLoS Comput Biol. 2012 Nov 1;8(11):e1002755. doi: 10.1371/journal.pcbi.1002755 (PMC3486846; doi:10.1371/journal.pcbi.1002755)
Supplement: Table S9 — Flux ratios (mean and median values) for a recovery factor of 0.5, using both the old and new estimation methods. Ribosomal densities were smoothed for all profiles using a window of 5–30 codons. (DOCX) [file pcbi.1002755.s026.docx]

|  | Flux ratio $\left( dx_{2}*\bar{D}_{2} \right)/({dx}_{1}*\bar{D}_{1})$ | | | |
| --- | --- | --- | --- | --- |
| Window size | Old method | | New method | |
|  | Mean+/-std | Median ratio | Mean+/-std | Median ratio |
| 5 | 39+/326 | 2.47 | 93+/461 | 4.36 |
| 10 | 32+/172 | 1.22 | 41+/187 | 2.82 |
| 15 | 19+/97 | 1.1 | 35+/174 | 2.06 |
| 20 | 31+/302 | 1.16 | 65+/533 | 1.93 |
| 25 | 40+/632 | 1.15 | 36+/202 | 1.87 |
| 30 | 18+/193 | 1.22 | 37+/203 | 1.69 |
