# Supplementary material for: Determinants of Translation Elongation Speed and Ribosomal Profiling Biases in Mouse Embryonic Stem Cells
Source: PLoS Comput Biol. 2012 Nov 1;8(11):e1002755. doi: 10.1371/journal.pcbi.1002755 (PMC3486846; doi:10.1371/journal.pcbi.1002755)
Supplement: Text S1 — Supplementary methods. (DOCX) [file pcbi.1002755.s028.docx]

Supplementary Text S1: Supplementary material for the paper 'Determinants and biases of translation elongation speed in mouse embryonic stem cells'

Alexandra Dana and Tamir Tuller

The Department of Biomedical Engineering, Tel-Aviv University, Tel-Aviv 69978, Israel

**Contact:** [**tamirtul@post.tau.ac.il**](mailto:tamirtul@post.tau.ac.il) **(TT)**

## Sources for reconstructing the *M. musculus* ribosomal profiles

The fragments used for reconstructing the ribosomal profiles of the isoforms analyzed in this study were taken from the GEO database (accession number GSE30839). Table S1 summarizes the specific file sources containing these fragments.

## Alignment statistics

Table S2 summarizes the alignment statistics of the processed fragments presented in Table S1, when aligned to the *M. musculus* exons (for more details about this procedure, see Methods). The first column represents the processed files, as defined in Table 1. The second column contains the number of read fragments from each one of the sources. The third column shows the number of valid fragments further processed after removing the linker (see Methods). The fourth column shows the percentage of data that was aligned to isoforms transcripts (exons and spliced junctions). The fifth column shows the percentage of the data that was aligned to a single location on isoforms transcripts. Columns six to eight show the percentage of data that was aligned to two/three/and more than three locations.

## The aggregated ribosomal density profile – using real measurements and simulation

Figure S1 shows the reconstructed ribosomal profiles (average view) of all isoforms with at least 1000 codons and a satisfactory number of reads (as published in Ingolia et al. [1]), for different run-off times. Each isoform profile was smoothed using a moving average window of 10 codons and normalized by its mean 800-1000 codon ribosomal reads. All normalized profiles were then averaged with equal weight.

Figure S2 presents the simulated ribosomal profiles of the genes presented in Figure S1, using the TASEP model (see Method). In the simulation, the translation velocity of each codon was defined according to its tAI value, using a low translation initiation rate (a tenth of the slowest codon). Run-off times were defined at $[150\Delta, 200\Delta, 250\Delta, 300\Delta]$, where $\Delta$ was set as the maximal translation time of a codon, according to the tAI measure (see Methods for more details). These run-off times were selected to resemble the reconstructed profiles presented in Figure S1. All simulated ribosomal densities profiles were normalized by their 800-1000 codon ribosomal densities and averaged with equal weight.

## Validating the assumptions made in the original *SL* estimation method

In the *original* work [1], ${RC}_{t}^{i}$ profiles were smoothed using an average window of five codons and then normalized by the mean read counts of codons 800-1000. In this study we wanted to validate if the read counts variability for codons 730-1000 is small enough (*i.e* ribosomal read counts in this region are similar), to support such scaling method.

For the validation, we analyzed all genes having profiles with sufficient read counts (see Methods) and longer than 750 codons. For each gene the read counts variability (standard deviation) was calculated using codons 730-1000. To allow comparison among genes, the STD was further normalized by the mean read counts for each gene apart. The results of this analysis are presented in Figure S3-A (blue histogram), resulting in a mean normalized STD of 1.1. For comparison purposes we also calculated the read counts variability of the ribosomal profiles of the analyzed genes, simulated with low (red), high (black) and proportional (green) initiation rates. The measured mean normalized STD on the simulative data was ~0.5 for all initiation rate regimes, indicating that for both the real and simulative profiles, the normalized standard deviation of the read counts of codons 730-1000 is significantly higher than 0. Therefore in general, ribosomal profiles cannot be normalized by using scaling factors that derive from averaging read counts selected from specific coding regions.

In addition, in the original method normalized run-off profiles were compared to a static threshold of value of 0.5. This assumed that baseline profiles have relatively similar read counts (when ignoring the first 40 codons and last 20 codons) and local noises could be filtered by smoothing the profiles. To validate this assumption, we applied the same calculation on all analyzed genes, when considering all their codons read counts (except the first 40 and last 20 codons). The results of this analysis are presented in Figure S3-B, indicating that also in general, the normalized variability of the read counts of all analyzed genes is not constant, but location dependent. Therefore, *SL* points cannot be determined by comparing the run-off profile to a static threshold.

## Measuring estimation errors for the original and the new suggested estimation method

Figures S4-S6 show the estimation error rates of the methods used for inferring the *SL* points as function of various parameters (noise level, recovery factors and smoothing windows) on data simulated under different initiation rate regimes. Both the original method presented in [1] and the new suggested method were tested on simulative data with different noise levels. Ribosomal densities of 100 different genes were created using the TASEP model (for more details see Methods). Codons translation efficiency was simulated based on the tAI measure and initiation rate was set to low (10% of the slowest possible codon), high (twice the fastest possible codon), or proportional to measured ribosomal read counts (for more details see Methods). The *SL* estimation methods were tested for different uniformly distributed noise levels added to the simulative profiles. The maximal noise level was set to be U ~[-¼, ¼]*max[ribosomal density of a gene]. Estimation error was defined as the distance between the *SL* points measured on the created ribosomal densities with zero noise levels and the relevant noise level. *SL* points for each analyzed gene were estimated for run-off times of [20$\Delta$, 50$\Delta$, 80$\Delta$, .. 200$\Delta$] time units, for a recovery factor of 0.5. The mean and standard deviation of the estimation error was calculated using all estimated *SL* points of all genes and *SL* points for a specific noise level. Each sub-figure presents the estimation error mean and STD for ribosomal density profiles smoothed with different window lengths (5/10/15/20/25/30 codons) prior to estimation. For all tested scenarios the new method results in lower estimation errors, suggesting a better robustness to noise. The estimation error also decreased as function of the length of the smoothing window.

## *SL* points estimated using the original and new estimated method – a visual comparison example

In the original method [1], prior to estimating the *SL* points, the run-off profiles of each analyzed isoform were scaled by normalizing the run-off profile with its codons' 800-1000 mean read counts (see Methods and Figure S3). In the new method, run-off profiles were scaled to the baseline profile by using a factor set to minimize the absolute distance between the two, without taking into consideration the first 730 and last 20 codons. All profiles were smoothed using an averaging window of 30 codons prior to scaling. Figure S7 presents the scaling results of the new method for the uc007gge.1 isoform. The baseline $RC_{0}$ profile is depicted in black, while the run-off profiles for 90/120/150 s are described in blue/red/green accordantly. In the original method, the estimation of the *SL* point was defined as the point where the run-off profile exceeded half of the baseline profile. These *SL* point estimations are presented in this example using a cyan/magenta/dark green dotted line. In the new suggested method, a *SL* point was defined as the location where the run-off profile exceeded the baseline profile (gray dotted curve) multiplied by a recovery factor (in this example it was set also to 0.5). *SL* points locations estimated using this method are presented in Figure S7 with blue/red/green dotted lines. The results show that the original method estimates *SL* points earlier than the new method, due to sensitivity to local noises, problematic profiles scaling and static threshold usage.

## Estimating *SL* points using different methods and estimation parameters

This section provides a comparison between the *SL* points estimated using the original and new method, as factor of the window size used to smooth the profiles and as factor of the recovery factor, used for multiplying the baseline profile (Column 1). Specifically, Columns 2-4 describe the average and standard deviation of the location of the estimated *SL* points, in codon units. Columns 5-6 present the average translation velocities, $v_{2}$ and $v_{1}$ in each segment. Column 7 provides a comparison between the estimated velocities $v_{1}$ and $v_{2}$, by using a p value calculated using the Kolmogorov-Smirnov test (ks-test). Columns 8 shows the average translation velocity defined by estimated $v_{1}$ and $v_{2}$, while Column 9 shows the median value of the ratio between translation velocities $v_{2}$ and $v_{1}$, calculated for each analyzed gene. Column 9 presents the median value of the difference between translation velocities $v_{1}$ and $v_{2}$ ${|v}_{1}-v2|/min(v_{1},v_{2})$ calculated per gene. Column 10 presents the number of analyzed isoforms that their *SL* points satisfied $x_{1}<x_{2}<x_{3}$. Tables S3-S4 show the above values based on *SL* points calculated using the original method, while Tables S5-S6 show the above values based on *SL* points calculated using the new suggested method.

## Explaining the differences in segments length using various properties of the coding sequence

To explain the differences between the lengths of the $dx_{1}$, $dx_{2}$ segments, all segments were sorted according to their genes' tAI/CAI values/segments folding energy/charge. Segments associated with top and bottom 20%/30%/40%/50% values according to these features were selected and their average and mean velocity was calculated. Figure S8 describes the result of this division, while Table S7 shows the calculated p-values between the samples of each two groups, for the different features, using an unparied t-test and Kolmagorov-Smirnof tests.

The results indicate that segments associated with high tAI/CAI (top 20%) were found to be significantly faster when compared to segments with low tAI/CAI values (bottom 20%), (t-test: p<0.00049/0.014 respectively;). Significant results were also obtained when dividing the segments according to their folding energy, using the top/bottom 20% division, with strongly folded segments being more slowly translated (t-test: P = 0.0023; ks-test: 0.04). Finally, when the segments were divided according to their charge in a similar manner (top/bottom 20%), segments less positively charged were translated faster (t-test: P=0.0014; ks-test: 0.0019). Therefore, this analysis also indicates that genes with higher tAI/CAI values (more recognized by the tRNA pool) / segments less tightly folded / more negatively charged are associated with longer segments (more efficiently translated).

## Numerical comparison of the DSRC measure and flux ratio as function of the *SL* estimation methods

The DSRC measure and flux ratio were calculated for all analyzed genes, using *SL* points estimated using both the original and new method, for different values of their parameters. Table S8 presents the median value of the DSRC measure (column 2 and 4) and the Spearman correlation results between $\bar{D}_{2}/\bar{D}_{1}$ and $dx_{2}/dx_{1}$ (see Results for definitions; column 3 and 5) when using both estimation methods for different recovery factors. The results indicate that the median DSRC value is higher than the expected value of 0 (see simulation results in Results chapter), while the correlation between $\bar{D}_{2}/\bar{D}_{1}$ and $dx_{2}/dx_{1}$ is relatively small (in comparison to the expected high negative correlation, see simulation results in Results chapter), regardless of the used estimation method and recovery factor value.

Table S9 presents the ratio between the flux of the first and second segments $\left( dx_{2}*\bar{D}_{2} \right)/({dx}_{1}*\bar{D}_{1})$, calculated using *SL* points estimated using both the original and new method, for smoothing windows of different sizes (used to smooth the profiles). The results indicate that this measure results in values considerably higher than 1 (as expected under the biophysical assumptions, see Results), regardless of the used estimation method and the size of the smoothing parameter.

## Simulating ribosomes read count profiles using monosome covered fragments and low number of mRNA copies

To study the effect of the number of mRNA molecules on the DSRC measure and on the correlation between $\bar{D}_{2}/\bar{D}_{1}$and $dx_{2}/dx_{1}$, ribosomal profiles of the analyzed isoforms in this study were simulated using the biophysical TASEP model for different numbers of mRNA copies. In addition, ribosomal profiles were also constructed using fragments covered by monosomes only (for more details see Methods). All simulations were performed for low and high initiation rates and ribosome locations were defined by the profiles created at times [100$\Delta T, 160\Delta T, 220\Delta T]$.

Figure S9 shows the DSRC measure calculated on simulative profiles containing all fragments (left column) and only fragments originating from monosomes (right column). The ribosomal profiles for the upper/lower two images were created using a low/high initiation rates. The number of simulated mRNA molecules per isoform was set to 20. Figures S10 and S11 present the same analysis, based on ribosomal profiles created using 50 and 500 mRNA molecules. Overall, the results indicate that a low number of mRNA copies per isoform and the removal of fragments covered by polysomes can drastically increase the DSRC measure (by the biophysical assumption, this value is supposed to be zero, see assumptions in Results chapter) for both initiation rate regimes.

Figures S12-14 present in a similar manner the Spearman correlation between $\bar{D}_{2}/\bar{D}_{1}$ and $dx_{2}/dx_{1}$ measures, calculated on simulated ribosome profiles, created with low and high initiation rates (top/bottom), with all fragments (left columns) and only fragments covered by monosomes (right column), where each ribosomal profile was simulated using 20/50/500 mRNAs copies. The results also show that a low number of mRNA molecules (especially for the low initiation regime) and the removal of fragments covered by polysomes (especially for the high initiation regime) decrease the correlation between $\bar{D}_{2}/\bar{D}_{1}$ and $dx_{2}/dx_{1}$ (from the biophysical perspective, this correlation is supposed to be highly negative, see assumptions in Results chapter),.

## Simulating non-uniform effect of harringtonine on ribosomal profiles

The propagation time of harringtonine for each gene was simulated as a random variable of uniform distribution ~ U([0, K]), where K = [0,10$\Delta T$, 20$\Delta T$, .. 200$\Delta T$]. *SL* points were calculated on profiles simulated for run-off times of [150$\Delta T$, 200$\Delta T$, 250$\Delta T$, 300$\Delta T$], for a low initiation regime. Figure S15 presents the averaged simulation profile for K = 0/40$\Delta T$/90$\Delta T$/140$\Delta T$. As seen from the figure, profiles created with highly non-uniform harringtonine propagation times are more smeared, similar to the real measured profile, presented in Figure S1.

To calculate the bias caused by the non-uniform effect of harringtonine in the estimation process of the *SL* points, for each isoform *SL* points were calculated on the simulative profile for all values of K. The difference between the value of the *SL* points for K=0 and K>0 was calculated. Figure S16 (A) presents the average difference in estimations as function of K for all analyzed genes, while Figure S16 (B) presents the velocities ratio $v_{2}/v_{1}$ as function of K. Figure S17 shows the DSRC measure and Spearman correlation values between $\bar{D}_{2}/\bar{D}_{1}$ and $dx_{2}/dx_{1}$ under the effect of increasing K values. These results indicate that the non-uniform harringtonine propagation effect distorts the ribosomal profiles and increases the bias between the real and estimated real and estimated *SL* points locations, which eventually increase the DSRC measure and decrease the correlation between $\bar{D}_{2}/\bar{D}_{1}$ and $dx_{2}/dx_{1}$.

References

1. Ingolia, N.T., L.F. Lareau, and J.S. Weissman, Ribosome profiling of mouse embryonic stem cells reveals the complexity and dynamics of mammalian proteomes. Cell, 2011. 147(4): p. 789-802.
